# The more the better? Synergies of prosocial interventions and effects on behavioural spillovers^☆^

**DOI:** 10.1016/j.jeem.2024.103061

**Published:** 2024-11

**Authors:** Marius Alt, Hendrik Bruns, Nives Della Valle

**Affiliations:** a European Commission, Joint Research Centre, Via Enrico Fermi 2749, 21027 Ispra, Italy; b European Commission, Joint Research Centre, Rue du Champ de Mars 21, 1049 Brussels, Belgium

**Keywords:** Prosocial behaviour, Pro-environmental behaviour, Behavioural interventions, Policy mixes, Behavioural spillovers, Online experiment

## Abstract

Incentivising prosocial and pro-environmental behaviours is a sensitive endeavour. While behavioural change is urgently needed to mitigate the consequences of climate change, monetary interventions often have negative side effects. Such interventions are prone to motivation crowding, which can impede lasting positive behavioural change and stimulate negative temporal spillovers to other prosocial behaviours. In this study, we investigate whether implementing monetary interventions as part of policy mixes can mitigate these negative side effects. In an online experiment involving 3782 participants, we test whether the use of nudges that make personal and social norms salient can counteract the motivation-crowding effect and explore the effects of such policy mixes on temporal spillovers. We find that policy mixes of norm-based nudges and monetary incentives are more effective at stimulating engagement in targeted prosocial behaviour than no intervention when controlling for sample characteristics. Analysing the temporal spillover effects of these interventions reveals that policy mixes can alleviate the tendency of monetary incentives to negatively affect subsequent prosocial behaviour. This indicates that norm-based nudges are suitable complements to monetary interventions, facilitating long-lasting positive effects.

## 1 Introduction

To achieve climate neutrality goals, both societal change and technological advancement are essential (Devine-Wright et al., 2022). The latest report by the Intergovernmental Panel on Climate Change (IPCC) highlights the crucial role of people in climate action, emphasising the need for systemic and long-lasting change towards more sustainable lifestyles and behaviours (Shukla et al., 2022).

Monetary incentives can motivate people to behave more pro-environmentally (Maki et al., 2016). However, they have also been shown to crowd out people’s intrinsic motivation to do so (Cardenas et al., 2000, Rode et al., 2015). Monetary incentives for prosocial behaviours can actually decrease engagement in those behaviours by undermining individuals’ intrinsic prosocial^1^ motives (Frey and Jegen, 2001, Bowles, 2008, Bowles and Polania-Reyes, 2012). Motivation crowding occurs because monetary incentives reframe a previously prosocial decision as self-interested, result in long-term changes in endogenous preferences, reduce perceived autonomy, or convey specific information about the regulator (Bowles, 2008). Thus, they can also impact subsequent prosocial behaviours that are not intended to be influenced by the incentive, but which rely on the same intrinsic motives. We refer to this as a *behavioural spillover*.

Nudges, commonly defined as any aspect of the choice architecture that systematically influences behaviour without forbidding any options or significantly changing incentives (Thaler and Sunstein, 2008), can serve as viable alternatives or complements to monetary interventions (Loewenstein and Chater, 2017). They are perceived as less autonomy threatening than certain types of economic incentives (Bruns and Perino, 2023), and are overall less likely to result in motivational crowding-out (Gråd et al., 2021). However, there is evidence that nudges are not always effective (Nisa et al., 2019, Szaszi et al., 2022), or are less effective in real-world settings than in laboratory settings (DellaVigna and Linos, 2022), and their effectiveness has been shown to differ across various types of samples (Osbaldiston and Schott, 2012, Abrahamse and Steg, 2013, Niemiec et al., 2020, Bryan et al., 2021).

To address the limitations of monetary incentives and nudges (i.e., crowding-out, negative spillover and limited effectiveness) and leverage their strengths (i.e., effectiveness and limited crowding-out), a combination of both approaches can be implemented as a policy mix (Drews et al., 2020). A recent meta-analysis on policy mixes targeting pro-environmental behaviours suggests that, on average, policy mixes are more effective in promoting the desired behaviour than are monetary interventions implemented in isolation (Alt et al., 2024). Policy mixes also cater to the need for social and environmental policies to address multiple objectives simultaneously (van den Bergh et al., 2021). However, the available experimental evidence on policy mixes does not determine whether they persistently spill over to other behaviours. Spillover effects are unintended changes in subsequent and untargeted behaviour that occur in the same context as a result of behaviour change interventions (Dolan and Galizzi, 2015, Nilsson et al., 2017, Galizzi and Whitmarsh, 2019). To determine the net effects of environmental policies, including policy mixes, it is crucial to take these effects into account, particularly when monetary incentives are included (Carrico, 2021).

This paper aims to advance our understanding of policy mixes in motivating pro-environmental behaviour and mitigating potential negative spillover caused by monetary incentives. Specifically, it assesses the effectiveness of a monetary reward, a nudge emphasising personal or social norms or a combination of a monetary reward and a norm-nudge in effectively motivating and sustaining prosocial behaviours. We focus on norm compliance^2^ induced by personal or social norm nudges, acknowledging the potential to crowd in prosocial motives (d’Adda, 2011).^3^ In particular, nudges can reframe a prosocial decision as an alignment with norms, activating the underlying motive for norm compliance and cultivating a norm-focused mindset (Sacconi et al., 2008). There is some evidence to suggest that norm-based nudges have positive spillover effects on subsequent behaviour (Capraro et al., 2019), both alone and when combined with other interventions, such as interventions increasing the observability of individual actions (Bolton et al., 2021). In the experiment, participants engaged in a real-effort task (RET) in which their effort translated into prosocial contributions. Participants randomly received either no intervention, a single norm nudge or financial reward or a combination of a norm-based nudge and a reward. After completing an unrelated filler task, participants had the opportunity to donate to a prosocial cause.

First, we find that a monetary reward both on its own and in combination with norm-based nudges increased prosocial performance in the initial behaviour. Second, both norm-based nudges on their own positively spilled over to the unrelated prosocial task. When norm nudges were combined with the reward, this positive spillover effect was larger than for the monetary reward on its own. We further distinguished different components of spillover effects to identify underlying mechanisms.

This paper is structured as follows: Section 2 describes the experimental design, the treatments, the tasks, the predictions, the sample and our approach to identifying spillover effects. Section 3 presents the results, initially discussing the interventions’ effects on behaviour in the RET and then examining the spillover effects on subsequent behaviour. Section 4 discusses and concludes.

## 2 Design and method

### 2.1 Design

Our design aims to experimentally investigate the spillover effects of policy mixes containing either personal or social norm nudges and monetary rewards. The experiment features two types of prosocial behaviours in which participants can engage: behaviour 1 and behaviour 2. Interventions target behaviour 1, whereas the spillover effect will be evaluated with respect to behaviour 2, which is thus the main outcome variable. Throughout the experiment, participants can earn and spend “survey points”.^4^

**Fig. 1:**
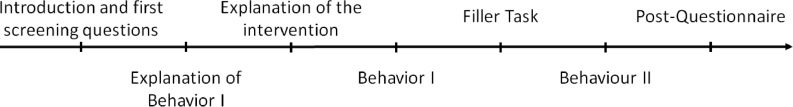
Structure of the experiment.

Fig. 1 outlines the experimental procedure. First, we introduce participants to the experiment and ask them four screening questions about their gender, age, education level, and region of residence.^5^ Second, we explain behaviour 1 to all participants and describe the relevant intervention to those assigned to an intervention treatment. After participants engage in behaviour 1, they take part in a filler task for the purpose of distraction. Participants then engage in behaviour 2, following which they are asked to complete a questionnaire comprising 20 questions about personal characteristics, beliefs and preferences. In the following sections, we explain the two prosocial behaviours and the treatment interventions and address how we adapted the design to avoid endogeneity of the measurement.

### 2.2 Behaviour 1

Behaviour 1 consists of participants’ actions in an RET. The RET allows participants to translate effort into charitable donations, specifically by decoding digits into letters. This task allows us to control for heterogeneous ability to complete RETs (Dorner, 2019). Participants receive a decoding table with seven digits and seven letters, with each letter corresponding to a digit. Participants are then instructed to decipher the codes, that is to translate the seven-digit codes into letters. Each correct deciphering leads to a donation to a food bank.^6^

Importantly, the size of the donation varies randomly across participants between 0 and 12 points in steps of two (explained in more detail in Section 2.8).^7^ If the answer is incorrect, participants are notified and receive a new code to be deciphered. In total, participants have 6 min to solve as many codes as possible.

In addition to working on the decoding tasks, participants have the option to enter the “automation mode”. In this mode, participants can remain inactive for 6 min. This mode results in a donation amount 20 times smaller than the amount that is donated by breaking a code every 10 s. Hence, participants face a trade-off between the utility obtained from contributing to a public good while facing costs of effort provision and the utility obtained from consuming leisure. We implement this mode to induce larger heterogeneity in effort levels (Erkal et al., 2018). Participants begin with the decoding task, but can switch back and forth between the task and the “automation mode” as they please.

### 2.3 Treatment interventions

The treatment interventions for behaviour 1 are listed and briefly described in Table 1. They comprise the control treatment without intervention, the single interventions and the policy mixes.

The treatment *prosocial only* serves to assess the effect of interventions on behaviour 2. Here, effort in behaviour 1 is not externally incentivised, such that it relies only on intrinsic motives.

**Table 1:** Description of treatments.

Interventions	Description
Prosocial only	No intervention
Personal norm nudge	Elicitation of personal norms w.r.t. the behaviour in behaviour 1
Social norm nudge	Elicitation of beliefs about others’ behaviour in behaviour 1
Monetary reward	10 survey points for each correctly answered code
Policy mix: Mon. rew. x Pers. norm nudge	Elicitation of personal norms w.r.t. the behaviour in behaviour 1 AND 10 survey points for each correctly deciphered code
Policy mix: Mon. rew. x Soc. norm nudge	Elicitation of beliefs about others’ behaviour in behaviour 1 AND 10 survey points for each correctly deciphered code

The two *norm nudge* treatments rely on one of many different ways through which norm-based nudges can be operationalised (Bicchieri and Dimant, 2022): they make personal and social perceptions of appropriateness salient. This means, for social norms^8^ eliciting what others think one should do (Bicchieri and Dimant, 2022, Bolton et al., 2021), and, in the case of personal norms,^9^ eliciting individual beliefs about correct or moral behaviour (Capraro et al., 2019, Kurschilgen, 2023).

In the *personal norm nudge* treatment, we use the approach taken by Bašić and Verrina (2023):

*[W]e would like you to evaluate, according to your own opinion and independently of the opinion of others, which of the following numbers of codes would be appropriate to solve in the task. “Appropriate” behaviour means the behaviour that you personally consider to be “correct” or “moral”. The standard is, hence, your personal opinion, independently of the opinion of others.*

By allowing participants to reflect on their evaluation of correct behaviour in behaviour 1, we make their personal norms salient. Although the assessment of a moral behaviour will differ among individuals, it sets an endogenous norm to comply with behaviour 1 and thereby, might increase effort.

In the *social norm nudge* treatment, we employ the social norm elicitation task from Bašić and Verrina (2023):

*[W]e would like you to guess which of the following numbers of codes other participants rated as the appropriate number to solve in the task, independent of your own opinion on the appropriate behaviour. “Appropriate” behaviour means the behaviour that others consider to be “correct” or “moral”. The standard is, hence, the opinion of others, independently of your personal opinion*.^10^

Eliciting beliefs about collective perceptions of appropriateness is incentive compatible (Krupka and Weber, 2013). If participants guess the beliefs of other participants about the appropriate number of codes solved in behaviour 1 correctly or with a divergence of 1, they receive 10 survey points.

In the *monetary reward* treatment, participants receive 10 survey points for each code correctly deciphered as a private payment. Given a positive marginal utility of income, this should increase their effort provision in behaviour 1.

The policy mix treatments combine either the personal or social norm nudge with the monetary reward. Participants first read the norm elicitation statement, and then are presented with information about the additional monetary reward. In this way, a participant’s stated personal norm remains comparable to the personal norms elicited in the *personal norm nudge* treatment.

### 2.4 The filler task

To distract participants from their actions in behaviour 1 and reduce experimenter demand, we implement a filler task between behaviour 1 and behaviour 2. This task consists of seven questions from cognitive reflection tests (Frederick, 2005, Thomson and Oppenheimer, 2016) and seven questions about a participant’s locus of control (Andor et al., 2022).^11^ On average, participants need about 3 min to answer all the questions.^12^

### 2.5 Behaviour 2

Behaviour 2 consists of a donation task in which participants can choose to donate part of their remuneration to a charitable organisation. Participants’ endowments (funds available for donation) at this point vary depending on their prior performance and the assigned treatment. Therefore, we limit the maximum amount that can be donated. This assures that the maximum amount remains affordable for each participant. Participants can donate to *Solar Aid*, *the Red Cross*, *Doctors without Borders*, *Save the Children* or *the World Wide Fund for Nature*. To ensure sufficient variation in the amounts donated to the selected charity, we match the donation of participants on a one-to-one ratio.

### 2.6 Endowment effects and behaviour 2

In behaviour 2, the monetary remuneration obtained throughout the experiment up to this point can affect donation amounts through income effects (de Araujo et al., 2015).^13^

To assure identical maximum donation amounts in behaviour 2 (70 survey points), participants with insufficient funds following behaviour 1, which is to say from treatment groups that did not receive additional monetary rewards or in case of insufficient codes broken in the monetary rewards treatment, receive the missing difference before entering behaviour 2. This windfall profit is framed as remuneration for completing the filler task.^14^

Table 2 shows the mean earning prior to behaviour 2 by treatment. Asterisks indicate a statistically significant difference from the treatment *prosocial only*. We observe similar values in the non-monetary reward treatments, whereas monetary rewards evidently lead to higher earnings (with all p-values of Mann–Whitney-U tests <0.0001).^15^ We thus control for participants’ earnings in the regressions.

**Table 2:** Earnings prior to behaviour 2 by treatment.

Prosocial only	Personal norm nudge	Social norm nudge	Monetary reward	Mon.rew. × pers. norm.	Mon.rew. × soc. norm
109.71	107.1	109.85	119.69***	120.05***	121.88***
(34.26)	(32.2)	(34.96)	(41.08)	(41.31)	(41.98)

Note: The table reports average earnings by participants in survey points prior to behaviour 2 across treatments. Standard deviation in parentheses. ${}^{+}$p < 0.1; ${}^{*}$p < 0.05; ${}^{**}$p < 0.01; ${}^{***}$p < 0.001.

### 2.7 Predictions

**Fig. 2:**
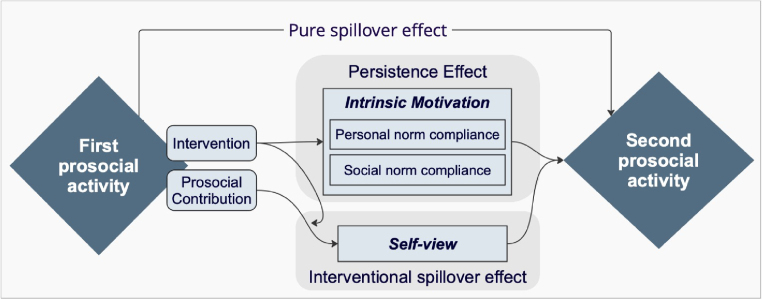
Utility framework of spillovers. *Note:* The figure shows the utility framework, indicating the channels through which the first prosocial activity affects the second prosocial activity. The grey boxes represent the *overall spillover effect* components.

To analyse spillover effects of policy mixes, in the following, we derive testable predictions.^16^ The framework, presented in Fig. 2 and outlined in detail in Supplementary material, Section2.2, consists of three main utility components: (1) the intrinsic motive for personal norm compliance, (2) the intrinsic motive for social norm compliance, and (3) self-view. Like the experiment, the framework consists of two periods, in both of which agents can engage in a prosocial activity. In the first period, this activity can be targeted by different interventions. We assume that spillovers from the first prosocial activity affect the marginal utility of engaging in the second prosocial activity. In particular, there are two types of spillover effects: the *pure spillover effect* and the *overall spillover effect*. The latter consists of the *persistence effect* and the *interventional spillover effect*.

The *pure spillover effect* reflects how engaging in a prosocial activity in one period affects the marginal utility of engaging in a subsequent prosocial behaviour, independent of any intervention (Lacasse, 2016, Lauren et al., 2019). Prosocial actions in the first period can either encourage continued prosocial behaviour (foot-in-the-door effect) or provide a licence for less prosocial behaviour (Monin and Miller, 2001, Mazar and Zhong, 2010, Bénabou and Tirole, 2011b). Aligned with findings from meta-studies by Blanken et al. (2015) and Maki et al. (2019), we propose that prosocial actions improve an agent’s self-view, in turn licensing decreased motivation for further prosocial actions. **Prediction 1.** *The pure spillover effect is negative.* The *overall spillover effect* reflects how interventions influence the marginal utility of prosocial activity in the second period. It consists of the *persistence effect* and the *interventional spillover effect*.

The *persistence effect* captures how the intervention affects the intrinsic motivation and subsequently the marginal utility of behaviour 2. The agent’s intrinsic motivation consists of the personal norm and the social norm compliance motive. Personal norm compliance captures the motivation of people to engage in a prosocial action to adhere to their personal norms (Festré, 2010, Bašić and Verrina, 2023).^17^ Social norm compliance captures the motivation to engage in a prosocial action to adhere to social norms (Bénabou and Tirole, 2006, Graf et al., 2023).^18^ An intervention targeting a prosocial behaviour can directly affect the motivation to engage in a subsequent prosocial activity (d’Adda, 2011). Thus, the *persistence effect* reflects how interventions directly affect the intrinsic motive for personal and/or social norm compliance (d’Adda et al., 2017). It thereby captures how an intervention targeting a prosocial activity affects the marginal utility of a subsequent prosocial activity, regardless of the prosocial contribution in the first activity (Alt and Gallier, 2022).

The *interventional spillover effect* influences the marginal utility of the second prosocial task through the agent’s self-view. Self-view refers to the need to build a positive self-image, which provides utility to the agent. It depends on, among other things, past prosocial behaviours (Bénabou and Tirole, 2011b).^19^ The *interventional spillover effect* is based on the assumption that a prosocial contribution in the first period is perceived differently depending on whether and how it has been incentivised (Bénabou and Tirole, 2011a, d’Adda et al., 2017). Thus, the intervention effect spills over, since the altered perception of the prosocial contribution affects the agent’s self-view, which again affects the marginal utility of a second prosocial activity.

In the following, we analyse the spillover effect of the interventions tested in the experiment, that is, a monetary reward and personal and social norm nudges, in accordance with our utility framework.

We start with the spillover effect of a **monetary reward** on personal norm compliance and social norm compliance. Based on results from previous studies (Rode et al., 2015, Chao, 2017, Bruni et al., 2020), we assume that personal norm compliance and monetary rewards can serve as substitutes (Bowles and Hwang, 2008). Thus, we predict monetary rewards to diminish the marginal utility of personal norm compliance. Similarly, we assume that monetary rewards for a prosocial activity crowd out the motive for social norm compliance (Rode et al., 2015, Kerr et al., 2019). Therefore, we expect a negative *persistence effect*.^20^

Regarding the spillover effect channelled through one’s self-view, we assume that monetary interventions increase the need to further invest in self-view. This is based on evidence that monetary rewards dilute the positive signal derived from engaging in prosocial behaviour for the agent’s self-view (Gneezy et al., 2011, Mullen and Monin, 2016, Ling and Xu, 2021), requiring further affirmation in the subsequent prosocial activity. Thus, we expect a positive *interventional spillover effect*.

As the relative magnitude and the direction of the different spillover effects require empirical validation, we illustrate one possible scenario, one that also features prominently in the literature (Maki et al., 2019, Alt and Gallier, 2022), namely assuming that monetary incentives adversely affect the marginal utility of engaging in subsequent prosocial activities, leading to a negative *overall spillover effect*.

Regarding the spillover effects of **personal and/or social norm nudges**, we assume that they work by increasing the intrinsic motive for personal norm (Capraro et al., 2019) and social norm compliance (Bolton et al., 2021), respectively. This effect is initiated when the personal/social norm nudge makes personal/social norms more salient in the first period, thereby positively spilling over to the second period. Thus, for both nudges, we expect a positive *persistence effect*.

Regarding the spillover effect obtained from self-view, we predict a mechanism similar to that for monetary rewards. By suggesting that past prosocial behaviour is a response to the personal/social norm nudges, interventions make it less likely that past prosocial behaviour is seen as an investment in self-view (Gneezy et al., 2011, Mullen and Monin, 2016, Ling and Xu, 2021), enhancing the need for further investments in self-view. Thus, we expect a positive *interventional spillover effect* for the social and personal norm nudges. Similarly to monetary rewards, the two types of spillover effects (i.e. persistence and interventional) point in opposite directions. Thus, determining the *overall spillover effect* remains an empirical task.

Next, we present the spillover effects of the policy mix including a **personal norm nudge and monetary rewards**. We do so by examining changes in the marginal utility of prosocial activities due to these interventions. For simplicity, we assume that these effects are additive.

Regarding the *persistence effect* of this policy mix, we predict that it will have a positive influence on the engagement in the second prosocial activity, since the personal norm nudge enhances the personal norm motive. This is expected to counteract the negative *persistence effect* of monetary incentives. Therefore, a policy mix involving a personal norm nudge and a monetary reward is expected to yield a larger persistence effect than a monetary reward alone.^21^ **Prediction 2a.** *The persistence effect of policy mixes comprising a personal norm nudge and a monetary reward will exceed the persistence effect observed for monetary rewards alone.* Regarding the *interventional spillover effect*, both the personal norm nudge and the monetary reward dilute the positive signal to one’s self-view. This increases the need for reaffirmation of prosocial signals in the second period, thereby enhancing the marginal utility of engaging in subsequent prosocial activities beyond what a monetary reward alone would achieve: **Prediction 2b:** *Policy mixes comprising a personal norm nudge and a monetary reward will generate a more positive interventional spillover effect than monetary rewards alone.* When considering the *overall spillover effect*, we thus observe that both the *persistence effect* and the *interventional spillover effect* increase the marginal utility of the subsequent prosocial activity more strongly than monetary rewards alone. This leads to our next prediction. **Prediction 2c.** *Policy mixes combining a personal norm nudge and a monetary reward will yield a larger overall spillover effect than using monetary rewards alone.* Next, we analyse the spillover effect of the policy mix comprising **a social norm nudge and monetary rewards**. We expect the negative effect of monetary rewards on the intrinsic motive for social norm compliance to be alleviated by the positive effect of the social norm nudge on this motive. Therefore, a policy mix involving a social norm nudge and a monetary reward is expected to yield a larger *persistence effect* than monetary rewards alone. **Prediction 3a.** *The persistence effect of policy mixes comprising a social norm nudge and a monetary reward will exceed the persistence effect observed for monetary rewards alone.* Regarding the *interventional spillover effect*, both interventions affect the agent’s self-view in a similar manner. Monetary rewards and social norm nudges dilute the first prosocial activity signal as a moral achievement, requiring additional investment in self-view in the second period. This need for investment is greater when using this policy mix than it is when using monetary rewards alone, leading to the following prediction. **Prediction 3b.** *Policy mixes comprising a social norm nudge and a monetary reward will generate a more positive interventional spillover effect than the use of monetary rewards alone.* When considering the *overall spillover effect*, we thus observe that both the *interventional spillover effect* and the *persistence effect* are predicted to be larger than the corresponding effects of monetary rewards alone. This leads to the following prediction. **Prediction 3c.** *Policy mixes combining a social norm nudge and a monetary reward will yield a larger overall spillover effect than the use of monetary rewards alone.*

### 2.8 Identification strategy and spillover effects

In the following, we explain how we identify the different spillover effects in the experiment. A detailed explanation of the identification strategy can be found in Supplementary material, Section 2.1.

The *overall spillover effect* measures how the donations in behaviour 2 are affected by performing behaviour 1 under a certain intervention. We identify the *overall spillover effect* by contrasting donations in behaviour 2 in the *prosocial only* treatment with donations in the treatments, in which an intervention is imposed on behaviour 1.

The identification of the *pure spillover effect* and the *interventional spillover effect* is less straightforward. The *pure spillover effect* measures the level of donations in behaviour 2 affected by the level of prosocial contribution achieved in behaviour 1 without any intervention. The *interventional spillover effect* captures how the donations in behaviour 2 differ depending on the prosocial contribution and the imposed intervention in behaviour 1. Thus, the identification strategy for the *pure* and *interventional spillover effects* must rely on the correlation between behaviour 1 and behaviour 2. However, this correlation is likely to be driven by endogenous factors. In particular, some omitted variables, such as personal characteristics and preferences, might determine donation decisions in behaviour 1 and behaviour 2. Therefore, we rely on an approach that introduces exogenous variation in contributions in behaviour 1 and use an instrumental variable approach to estimate the *pure spillover effect* and the *interventional spillover effects*. We implement exogenous variation in the contributions to charity in behaviour 1 by randomly assigning different values donated by participants once they break a code. These values range from 0 to 12 survey points in steps of two. We use this variable in a two-stage least squares (2SLS) regression design, in which we regress the total donations generated by breaking codes within the 6 min of behaviour 1 on the specific donation value assigned to participants for breaking a code in behaviour 1. This reflects our 2SLS’s first stage. We use the fitted values from this regression as an instrument in the second stage. We argue that the instrument fulfills the exclusion restriction, as the exogenously assigned donation in behaviour 1 can affect the donation decision in behaviour 2 only through the spillover effect. In the second stage, we estimate the correlation between the effect of the exogenously imposed variation in the donations in behaviour 1 and the corresponding effect on behaviour 2. Thus, this estimate is independent of endogenous factors.

Finally, the *persistence effect* measures how donations in behaviour 2 depend on the intervention in behaviour 1 given no prosocial contribution is achieved. Here, we make use of the random assignment of values donated by participants once they break a code in behaviour 1. By assessing only the subset of participants, who are assigned a donation value of zero, we are able to uniquely assess how the intervention spills over. Thus, by comparing the donations of participants who were subjected to an intervention but did not make a prosocial contribution in behaviour 1 with the donations of those who were not subjected to an intervention and did not make a prosocial contribution in behaviour 1, we obtain the *persistence effect*. Due to endogeneity concerns, we perform this analysis within the abovementioned 2SLS design.

### 2.9 Procedure and sample characteristics

The experiment was conducted online on behalf of the authors’ institution by Ipsos, a global research and survey company, as part of a consortium with LE Europe.^22^ The experiment was carried out in February 2023 with participants from Italy and Poland.^23^ The sample size for each treatment and country is shown in Table 3.^24^

The programming of the experiment was carried out by Ipsos in close liaison with the authors and based on a detailed script written by the authors (see Supplementary material, Section 3).

**Table 3:** Sample size per experimental condition and country.

Treatment	Total sample size	Italy	Poland
Prosocial only	784	381	403
Personal norm nudge	584	296	288
Social norm nudge	584	287	297
Monetary reward	614	308	306
Monetary rew. & pers. norm nudge	612	312	300
Monetary rew. & soc. norm nudge	604	303	301

Total	3782	1887	1895

Table 4 shows the distribution of descriptive sample variables across treatments. On average, participants were about 49 years old with 13 years of education. Around 53% of the sample were female. To better disentangle the effect of our treatments, we control for key economic preferences (shown in Table 4) in regressions. These are common predictors of public good contributions (Lades et al., 2021).

**Table 4:** Sample characteristics.

Age	Female	Education	Altruism	Past donations	Time preferences	Trust	Risk
48.96	0.53	13.07	1.76	1.78	2.62	1.7	5.16
(16.72)	(0.5)	(2.42)	(1.4)	(1.47)	(0.87)	(0.74)	(1.93)

*Note:* Standard deviation in parentheses. The variables have been elicited in the post-questionnaire. *Age* and *education* are measured in years; *altruism* is measured on a scale from 0 (not willing to give) to 4 (very willing to give); *past donations* state whether participants did not donate (coded as 0), donated less than EUR 10 (coded as 1), between EUR 10 and EUR 50 (coded as 2), or more than EUR 50 (coded as 3) in the year prior to the experiment. *Time preferences* are measured on a scale from 1 (unwilling to give up current benefits) to 5 (very willing to give up current benefits). The scale for trust also ranges from 0 (not trusting at all) to 4 (very trusting), while risk measures where participants fall on a range from “fully risk averse” (coded as 0) to “fully risk seeking” (coded as 10). Further information on the measurement of the variables can be retrieved from the scripted experiment (Supplementary material, Section 3).

## 3 Results

We present first the effects on behaviour 1, followed by the spillover effects on behaviour 2.

### 3.1 Interventions and performance in behaviour 1

**Fig. 3:**
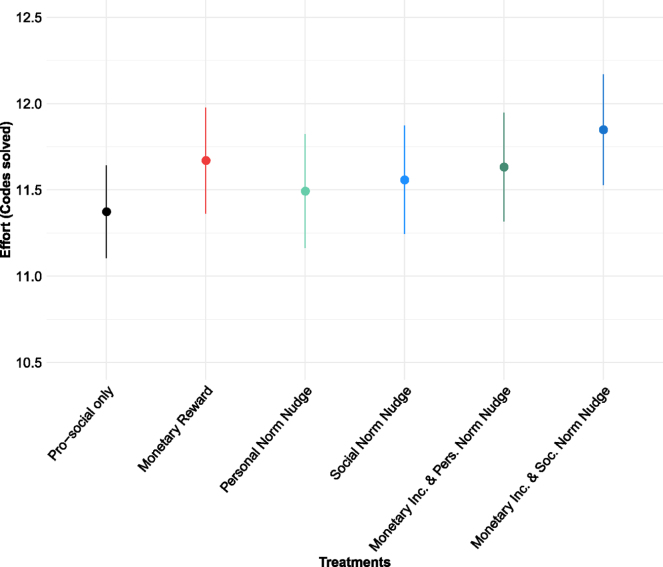
Effect of interventions on performance in behaviour 1. *Note:* The figure shows raw data means of effort provided in behaviour 1 across treatments. Effort is measured as the number of codes deciphered within the 6 min of behaviour 1. Error bars show confidence intervals at the 90% level. There are no differences between countries regarding the between-treatment effects, as shown in Table 9 of the Supplementary material.

Fig. 3 shows the average number of codes deciphered within the 6 min of behaviour 1 by treatments. Mean values in all intervention treatments are above the levels in the *prosocial only* treatment, but the differences are not significant. When including control variables in the regression model, performance in all treatments with monetary rewards is significantly higher than in the *prosocial only* treatment (*monetary reward*: p = 0.0317; *monetary reward* x *personal norm nudge*: p = 0.0281; *monetary reward* x *social norm nudge*: p = 0.0165, Table A.3 - column 3 in Appendix A).^25^ This suggests that, when controlling for sample characteristics, only interventions offering monetary rewards increase performance.

### 3.2 Interventions and spillover effects

To analyse spillovers on behaviour 2, we analyse first the *overall spillover effect*, then its components: the *interventional spillover effect* and the *persistence effect*.

#### 3.2.1 Overall spillover effects

**Fig. 4:**
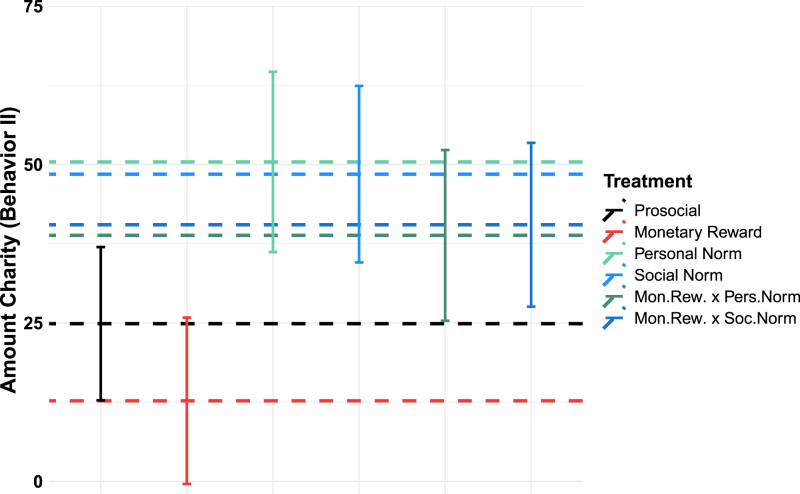
Donation levels in behaviour 2 across treatments. *Note:* Dashed lines show the tobit-regression coefficients and their linear combinations from Table A.3, column 1 in Appendix A. The y-axis depicts the effect of treatment interventions on donations (behaviour 2). The x-axis depicts the different treatments. The dependent variable is the amount donated in behaviour 2 in survey points. Error bars show confidence intervals at the 95% level. There are no differences between countries regarding the between-treatment effects, as shown in Table 9 of the Supplementary material.

Fig. 4 shows average donation levels across treatments. We derive all estimates from a tobit regression model (see Table A.3 - column 1, in Appendix A) and Wald tests of corresponding coefficients and linear combinations of coefficients.^26^

Without any intervention, participants donate on average 25 points (p = 0.0242, see Table A.3, column 1 in Appendix A). We obtain the *overall spillover effect* by comparing this average with the donations of participants subjected to intervention treatments.^27^ We do not find evidence for a significant spillover of monetary rewards. The decrease in average donations by around half, to 13 (i.e. 25–12) points, is not significant (p = 0.2574; linear combination of coefficients from Table A.3, column 1 in Appendix A).

For donations on the intensive margin (i.e., among those who donate), we observe a significant reduction induced by the monetary reward (b = −9.9, p = 0.0033, see Table A.4, column 3 in Appendix A). Moreover, excluding participants who could not donate in behaviour 1 because they were randomly assigned a donation value of zero unveils a significant negative *overall spillover effect* (b = −28, p = 0.0179, see Table 1 in Supplementary material, Section 1). Therefore, in the following, we refer to the *overall spillover effect* of the *monetary reward* treatment as tending to be negative.

Regarding the norm treatments, eliciting the personal norm for behaviour 1 positively spills over to behaviour 2. The average amount donated to charity in behaviour 2 is twice that donated in *Prosocial only* treatment (p = 0.0242, see Table A.3, column 1 in Appendix A), ranging at 50 points. The effect is similar for the social norm nudge, with average donations in behaviour 2 of 48 points, significantly higher than donations in *prosocial only* (p = 0.0349, see Table A.3, column 1 in Appendix A).

The policy mix of a monetary reward and a personal norm nudge led to subsequent average donations of 39 points, not significantly different from the value in the no-intervention treatment (p = 0.2033, see Table A.3 column 1 in Appendix A). The policy mix including the social norm led to similar average donations of 40 points, equally insignificant (p = 0.1443, see Table A.3, column 1 in Appendix A).

We further investigate whether policy mixes alleviated the tendency of monetary rewards to negatively spill over to subsequent prosocial behaviour. Indeed, we observe that both policy mixes led to significantly larger donations in behaviour 2 than the *monetary reward* treatment (monetary reward x personal norm nudge, p = 0.0218; monetary reward x social norm nudge, p = 0.0125, see Table A.3, column 1 in Appendix A).^28^

Result 1 *The *overall spillover effects* for both policy mix interventions are significantly larger than they are for *monetary rewards* alone*.

#### 3.2.2 Interventional spillover effects

**Fig. 5:**
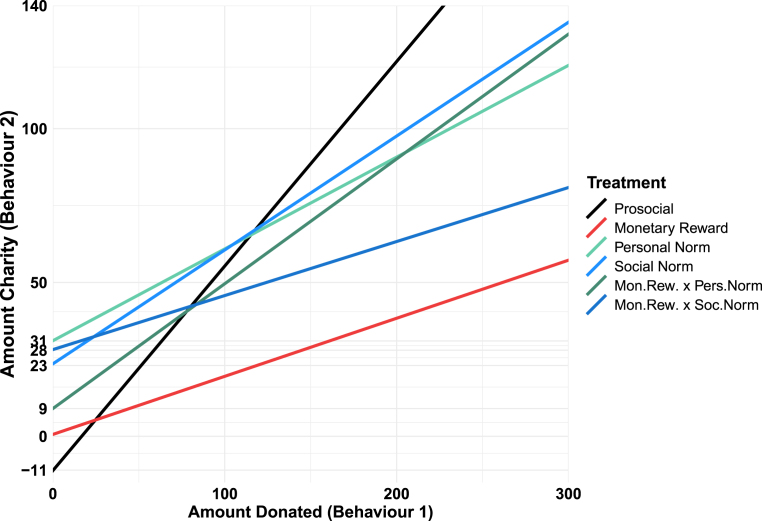
Spillover effects of interventions. *Note:* The lines show the interaction effects between the amount donated in behaviour 1 (in survey points), on the x-axis donations, and donations in behaviour 2, on the y-axis, for all treatments from the second stage of the 2SLS regression (where the first stage is ordinary least squares and the second stage is tobit), reported in Table A.3, column 1, in Appendix A. The dependent variable reports the amount donated in behaviour 2 in survey points.

To determine the *interventional spillover effect*, we scrutinise the relationship between behaviour 1 and behaviour 2. Fig. 5 illustrates this correlation based on fitted regression lines from a 2SLS tobit regression model (see Table A.3, column 2, in Appendix A). This correlation is based only on exogenous variation induced through the assignment of different donation values in behaviour 1. The effect in the *prosocial-only* treatment serves as the point of comparison for the *interventional spillover effects*. The respective fitted regression line of the *prosocial-only* treatment (in black) shows that each additional point donated in behaviour 1 increases the donations in behaviour 2 by 0.67 points on average (p < 0.0001, see Table A.3, column 2, in Appendix A). Thus, in contrast to prediction 1, we observe a positive *pure spillover effect*.

Result 2 *The pure spillover effect* is positive.

To identify *interventional spillover effects*, we compare the correlations between the *prosocial only* treatment and the intervention treatments separately. This reveals that an increase in the donations by 1 point induced through monetary rewards in behaviour 1 leads to a slight but insignificant increase in donations in behaviour 2 of 0.19 points (0.665–0.476) (p = 0.2491, see Table A.3, column 2, in Appendix A). Compared with the *prosocial only* treatment, this reveals a significantly lower correlation between behaviour 1 and 2 by 0.48 (p = 0.0303, see Table A.3, column 2, in Appendix A). This implies a negative *interventional spillover effect* for the *monetary reward* treatment.

For the *personal norm nudge* treatment, we observe a subsequent increase of 0.3 points (0.665–0.366), as a result of a 1-point rise in behaviour 1 donations (p = 0.1360, see Table A.3, column 2, in Appendix A). In contrast to the *Prosocial only* treatment, this reduction by 0.37 is not significant (p = 0.1399, see Table A.3, column 2, in Appendix A). Hence, there is no evidence for an interventional spillover effect caused by the personal norm nudge.

The same is the case for the *social norm nudge*, where a 1-point increase in donations in behaviour 1 causes an increase of 0.37 points (0.665–0.295) donated in behaviour 2 (p = 0.0315, see Table A.3, column 2, in Appendix A). Again, in contrast to *prosocial only* treatment, this difference is not statistically significant (p = 0.1922, see Table A.3, column 2, in Appendix A). Hence, there is also no evidence for an interventional spillover effect caused by the social norm nudge.

Lastly, we investigate the *interventional spillover effect* of the two policy mixes. For the policy mix involving a personal norm nudge, the correlation between donations is 0.41 points (0.665–0.259) on average (p = 0.0228, see Table A.3, column 2, in Appendix A). This is only 0.28 points lower than the correlation in the *prosocial only* treatment. Consequently, we observe no *interventional spillover effect* for the policy mix including a personal norm nudge (p = 0.2614, see Table A.3, column 2 in Appendix A).^29^ The contrast to the *Monetary reward* treatment is also not significant (p = 0.3695, Table A.3 - Column 2 in Appendix A). Therefore, we cannot confirm prediction 3b.

Result 3 The *interventional spillover effect* for the policy mix including a personal norm nudge is not significantly different from the *interventional spillover effect* of the monetary reward alone.

Similarly, the policy mix including the social norm nudge features an insignificant positive correlation between both behaviours of 0.18 points, 0.665–0.489 (p = 0.2993, see Table A.3, column 2, in Appendix A). This reflects a negative spillover of −0.49 compared with no-intervention (p = 0.0289, see Table A.3, column 2, in Appendix A). Compared with the group receiving a monetary reward, and contrary to prediction 2b, the policy mix containing a social norm nudge does not alleviate the negative *interventional spillover effect* induced by the single monetary reward (p = 0.9554, see Table A.3, column 2 in Appendix A).

Result 4 The *interventional spillover effect* for the policy mix including a social norm nudge is not significantly different from the *interventional spillover effect* of the monetary reward alone.

#### 3.2.3 Persistence effects

Interventions can also affect behaviour 2 independently of a prior prosocial contribution. This is the *persistence effect*, measured by analysing the intervention-induced change in behaviour 2 conditional on no donation in behaviour 1. In Fig. 5, this is provided by the difference in the intercepts of the fitted regression lines of the intervention treatments and the *prosocial treatment*.

While we observe no significant persistence effect for the monetary reward (p = 0.4910, see Table A.3, column 2, in Appendix A), the intercepts of the *personal norm nudge* and *social norm nudge* treatments are significantly larger than the intercept of the *prosocial treatment* by 42 (p = 0.0260) and 36 points (p = 0.0560), respectively (see Table A.3, column 2 in Appendix A). The policy mix including a personal norm nudge only modestly raises donations in behaviour 2 absent a prosocial contribution in behaviour 1. The corresponding *persistence effect* of 20 points is not significant (p = 0.2948, see Table A.3, column 2, in Appendix A). In contrast, the persistence effect of 39 points induced by the policy mix including a social norm is significant (p = 0.0319, see Table A.3, column 2, in Appendix A).

The comparisons between the *persistence effects* of the policy mixes and those of monetary rewards alone (0.08 points for the mix including a personal norm nudge and 28 points for the mix including a social norm nudge) are not significant either (p = 0.6795 and p = 0.1571 respectively; see Table A.3, column 2 in Appendix A). Thus, we cannot reject or confirm predictions 2a and 3a.

Result 5 *The *persistence effects* of both policy mixes are not significantly different from the *persistence effect* of the *monetary reward* alone.*

These results add to the explanation of the observed positive *overall spillover effects* for both policy mix treatments relative to the monetary reward, which are similar. However, the analysis of the *interventional spillover effect* and the *persistence effect* shows that the positive *overall spillover effect* is driven by a strong *interventional spillover effect* in the case of the policy mix involving the personal norm nudge. In contrast, the *monetary reward* x *social norm nudge* treatment’s *overall spillover effect* may be more driven by the *persistence effect*. An overview of all predictions and the corresponding findings is provided in Table 5.

**Table 5:** Hypotheses and findings overview.

Prediction	Finding	Test outcome
1 - $PSE_{\text{All}}<0$	2 - $PSE_{\text{All}}>0$	Not confirmed (opposite)
2a - $PE_{\text{PN+MR}}>PE_{\text{MR}}$	5 - $PE_{\text{PN+MR}}=PE_{\text{MR}}$	Not confirmed (null effect)
2b - $ISE_{\text{PN+MR}}>ISE_{\text{MR}}$	3 - $ISE_{\text{PN+MR}}=ISE_{\text{MR}}$	Not confirmed (null effect)
2c - $OSE_{\text{PN+MR}}>OSE_{\text{MR}}$	1 - $OSE_{\text{PN+MR}}>OSE_{\text{MR}}$	Confirmed
3a - $PE_{\text{SN+MR}}>PE_{\text{MR}}$	5 - $PE_{\text{SN+MR}}=PE_{\text{MR}}$	Not confirmed (null effect)
3b - $ISE_{\text{SN+MR}}>ISE_{\text{MR}}$	4 - $ISE_{\text{SN+MR}}=ISE_{\text{MR}}$	Not confirmed (null effect)
3c - $OSE_{\text{SN+MR}}>OSE_{\text{MR}}$	1 - $OSE_{\text{PN+MR}}>OSE_{\text{MR}}$	Confirmed

*Note:*PSE= pure spillover effect; PE= persistence effect; OSE= overall spillover effect; ISE= interventional spillover effect; SN= social norm nudge; PN= personal norm nudge; MR= monetary reward.

## 4 Discussion and conclusions

In an online experiment in which participants are on two occasions required to make prosocial decisions, but only the first of those occasions is subjected to an intervention, we find that policy mixes that include monetary rewards are more effective in promoting engagement in the targeted prosocial behaviour than no intervention, but only when controlling for covariates. With regard to spillover effects, we find that policy mixes alleviate the tendency of monetary rewards to negatively spill over to subsequent prosocial behaviour. This suggests that norm-based nudges can complement monetary rewards to ensure positive effects beyond the targeted behaviour.

The experimental set-up allows us to break down the *overall spillover effect* (a broad measurement of spillovers) into an *interventional spillover effect* and a *persistence effect*. Thereby, we aim to shed light on the mechanisms behind the two policy mixes. The policy mix of monetary reward and social norm nudge increases engagement in subsequent prosocial behaviour irrespective of actions taken in the targeted behaviour. This suggests that the increased salience of the social norm carries over to the untargeted prosocial behaviour, persisting over time.

The findings on the policy mix of a monetary reward and a personal norm nudge suggest that the latter intervention follows a more complex mechanism. The positive spillover of the personal norm nudge seems to depend on whether the stated personal norm is followed through in related behaviours. In the experiment, this implies that this policy mix successfully increases subsequent prosocial behaviour when the appropriate behaviour indicated as the personal norm is followed by a significant donation in the incentivised task. This suggests that personal norm nudges require a practical confirmation of a prior (personal norm compliant) prosocial behaviour.

At first glance, our findings challenge our predictions. In the case of the policy mix of a monetary reward and a social norm nudge, the identified interventional spillover effect is opposite to the predicted effect. However, this results from finding a negative *pure spillover effect*, contrary to our prediction of a positive one. This has implications for the *interventional spillover effect*. We theorised that interventions would dilute the signals from past prosocial behaviour, which should create a positive *interventional spillover effect* if the pure spillover effect was *negative*. However, the finding of a *positive* pure spillover effect suggests that interventions that dilute the signal from previous prosocial actions will have a negative *interventional spillover effect*, further weakening the already weak signal. Thus, assuming a *positive* pure spillover effect helps explain why we observe a negative *interventional spillover effect* in the treatment involving a monetary reward and a social norm nudge.

The findings offer some tentative insights for policymakers selecting and implementing environmental and climate policies. First, our results suggest that policymakers should consider potential spillover effects to capture the net impacts of their interventions. Second, monetary rewards for pro-environmental behaviour could crowd out intrinsic motivations, thus negatively spilling over to untargeted yet desirable behaviour. Considering factors of interventions that could drive negative spillover and persistence effects thus seems advisable. If monetary rewards are necessary, policymakers might want to consider accompanying them by norm nudges to alleviate potential negative spillovers. Reminding individuals of prevailing societal or personal beliefs regarding pro-environmental actions can counter potential negative spillovers induced by monetary rewards in certain circumstances.

There are certain caveats that need to be considered when interpreting the findings. In the experiment, the behaviours are temporally close to each other. Despite a filler task, the design is idealised. It is thus unclear to what extent our findings are generalisable to situations in which the two behaviours are temporally closer to or more distant from each other. As spillover effects decay with time (Schmitz, 2019), we expect a negative relationship between effect sizes and the time between behaviours. Additionally, our experiment focuses on charitable donations to a food bank, thus addressing a particular type of prosocial and pro-environmental decision-making. However, by demonstrating the negative interactions between intrinsic motives and economic incentives, it could reveal shared mechanisms underlying social and environmental preferences. Moreover, the interventions we use in the experiment are prototypes. Financial interventions to motivate pro-environmental behaviour can take a myriad of forms and levels. For example, rewards can be smaller or larger, or of another type, for example taxes or mandatory minimum contributions. The same applies to behavioural interventions eliciting personal and social norms. Other phrasings (more or less salient) can be used and other, non-text-based, interventions exist. Considering also that nudges can be effective but are highly context-dependent and often not persistent (Szaszi et al., 2018, Szaszi et al., 2022), it is thus crucial to carefully consider the scalability and long-term effectiveness of our interventions when drawing policy implications from our findings. Nevertheless, we think that the tested interventions capture key components and mechanisms. Lastly, spillover effects are hard to detect and usually suffer from small effect sizes (Blanken et al., 2015, Maki et al., 2019, Geiger et al., 2021). Although we have a relatively large sample in our study, many effects are not significant. Furthermore, it is not clear if these effects would also occur in a field setting with more participants. Since nudges implemented by governments can have a significant yet smaller impact than that observed in academic settings (DellaVigna and Linos, 2022), we encourage further validation of our results with larger numbers of observations, preferably in field settings.

In conclusion, our experiment is an initial step to better understand policy mixes and their mutual interactions regarding different components of behavioural spillovers. Thus, it sets the basis for further investigation and confirmation and offers initial guidance to climate and environment policymakers.

## Preregistration

The respective preregistration can be obtained from https://aspredicted.org/4P9_5HC.

## CRediT authorship contribution statement

**Marius Alt:** Writing – review & editing, Writing – original draft, Visualization, Methodology, Investigation, Formal analysis, Data curation, Conceptualization. **Hendrik Bruns:** Writing – review & editing. **Nives Della Valle:** Writing – review & editing, Writing – original draft, Validation, Supervision, Project administration, Methodology, Funding acquisition, Conceptualization.

## Declaration of Generative AI and AI-assisted technologies in the writing process

During the preparation of this work the authors used Chat GPT in order to improve readability and language. After using this tool, the authors reviewed and edited the content as needed and take full responsibility for the content of the publication.

## Declaration of competing interest

The authors declare that they have no known competing financial interests or personal relationships that could have appeared to influence the work reported in this paper.

## Figures and Tables

**Fig. A.1:**
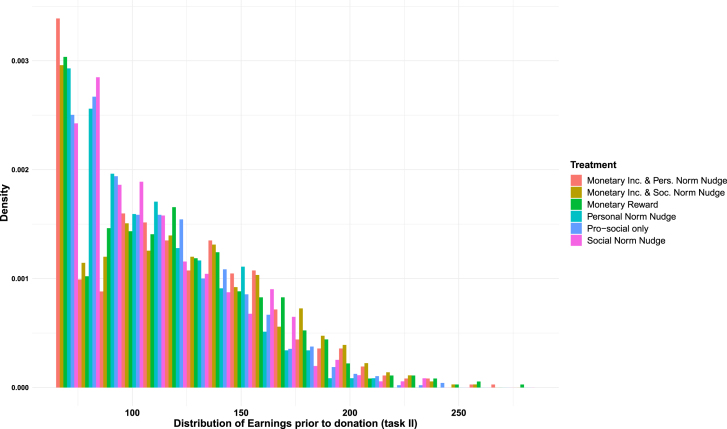
Earnings prior to behaviour 2 across treatments. *Note:* The figure illustrates the distribution of earnings of participants in our online experiment prior to behaviour 2 by treatments.

**Table A.1:** Sample characteristics by treatment.

	Pooled	Prosocial	Personal Norm	Social Norm	Monetary Reward	Mon.Rew.x Pers. Norm	Mon. Rew.x Soc. Norm
Age	48.96	49.27	49.76	49.84	48.3	48.22	48.31
	(16.72)	(16.87)	(16.88)	(16.79)	(16.36)	(16.48)	(16.87)
							
Education	13.07	13.09	13.05	13.27	12.95	13.2	12.85
	(2.42)	(2.39)	(2.31)	(2.43)	(2.43)	(2.54)	(2.41)
							
Female	0.53	0.53	0.54	0.53	0.54	0.53	0.52
	(0.5)	(0.5)	(0.5)	(0.5)	(0.5)	(0.5)	(0.5)
							
Altruism	1.76	1.74	1.74	1.79	1.66	1.82	1.83
	(1.4)	(1.38)	(1.42)	(1.37)	(1.42)	(1.49)	(1.34)
							
Past donations	1.78	1.86	1.81	1.75	1.79	1.68${}^{*}$	1.79
	(1.47)	(1.46)	(1.47)	(1.48)	(1.47)	(1.49)	(1.47)
							
Time preferences	2.62	2.58	2.59	2.68${}^{*}$	2.64	2.61	2.66
	(0.87)	(0.88)	(0.89)	(0.84)	(0.81)	(0.89)	(0.88)
							
Trust	1.7	1.7	1.71	1.64	1.67	1.76	1.71
	(0.74)	(0.73)	(0.75)	(0.75)	(0.7)	(0.74)	(0.76)
							
Risk	5.16	5.18	5.13	5.21	5.1	5.12	5.25
	(1.93)	(1.99)	(1.96)	(1.9)	(1.9)	(1.96)	(1.84)
							
Donation per code	5.6	4.56	5.62${}^{+}$	5.79	5.64	6.3${}^{+}$	6.05${}^{+}$
(Behaviour 1)	(4.13)	(4.29)	(3.82)	(4.22)	(4.17)	(3.87)	(4.1)

*Note:* Standard deviation in parentheses. Stars indicate difference to *Prosocial only* treatment: +p < 0.1; ${}^{*}$p < 0.05; ${}^{**}$p < 0.01; ${}^{***}$p < 0.001. The variables have been inquired in the post-questionnaire. *Age* and *Education* are measured in years; *Altruism* is measured on a scale from 0 (not willing to give) to 4 (very willing to give); *Past donations* state whether participants have not donated (coded as 0), donated less than 10 EUR (coded as 1), between 10-50 EUR (coded as 2), or more than 50 EUR (coded as 3) in the year prior to the experiment. *Time preferences* are measured on a scale from 1 (unwilling to give up current benefits) to 5 (very willing to give up current benefits). The scale for trust also ranges from 0 (not trusting at all) to 4 (very trusting), while risk measures whether participants are “fully risk averse” (coded as 0) up to “fully risk seeking” (coded as 10). Further information on the measurement of the variables can be retrieved from the scripted experiment, Supplementary Material, Section 3.

**Table A.2:** Performance in the decoding task by treatment.

	*Dependent variable:*		
	CodesSolved		
	(1)	(2)	(3)
Personal Norm	0.118	0.652	0.316
	(0.263)	(0.528)	(0.226)
			
Social Norm	0.189	1.385${}^{**}$	0.310
	(0.255)	(0.519)	(0.217)
			
Monetary Reward	0.315	1.215${}^{*}$	0.454${}^{*}$
	(0.250)	(0.481)	(0.211)
			
Mon.Rew. & Pers.Norm	0.272	1.225${}^{*}$	0.475${}^{*}$
	(0.255)	(0.492)	(0.217)
			
Mon.Rew. & Soc.Norm	0.478${}^{+}$	0.644	0.522${}^{*}$
	(0.258)	(0.512)	(0.218)
			
Constant	11.343${}^{***}$	11.000${}^{***}$	16.730${}^{***}$
	(0.166)	(0.316)	(0.684)
			

Below median altruists		×	
Control Variables			×
Observations	3,782	938	3,782
Log Likelihood	−11,167.650	−2,766.152	−10,509.280

*Note:* OLS regression. The dependent variable is given by broken codes within the six minutes in behaviour 1. In column 1 and 2, we use only the treatment indicators as independent variables. Column 2 repeats the column 1’s estimation on a restricted sample of participants who have a below median value in the altruism variable inquired in the post-questionnaire. Column 3 additionally controls for other covariates. The full regression table can be retrieved from the Supplementary Material, Table 1. Robust standard errors in parentheses. ${}^{+}$p < 0.1; ${}^{*}$p < 0.05; ${}^{**}$p < 0.01; ${}^{***}$p < 0.001.

**Table A.3:** Interventions and spillover effects.

	*Dependent variable:*			
	DonationsBehav2			
	(1)	(2)	(3)	(4)
Prosocial only			12.163	−11.756
			(10.741)	(17.067)
				
Personal Norm	25.530${}^{*}$	42.206${}^{*}$	37.694${}^{**}$	30.450
	(11.330)	(18.959)	(11.743)	(20.072)
				
Social Norm	23.595${}^{*}$	34.697${}^{+}$	35.759${}^{**}$	22.941
	(11.184)	(18.158)	(11.600)	(19.318)
				
Monetary Reward	−12.163	11.756		
	(10.741)	(17.067)		
				
Mon.Rew. & Pers.Norm	13.937	20.156	26.100${}^{+}$	8.401
	(10.956)	(19.240)	(11.374)	(20.335)
				
Mon.Rew. & Soc.Norm	15.615	39.326${}^{*}$	27.778${}^{+}$	27.570
	(10.697)	(18.333)	(11.122)	(19.486)
				
DonationsBehav1		0.665${}^{***}$		0.189
		(0.147)		(0.164)
				
Personal Norm × DonationsBehav1		−0.366		0.110
		(0.248)		(0.258)
				
Social Norm × DonationsBehav1		−0.295		0.181
		(0.226)		(0.237)
				
Monetary Reward × DonationsBehav1		$-0.476^{*}$		
		(0.220)		
				
Prosocial only × DonationsBehav1				0.476${}^{*}$
				(0.220)
				
Mon.Rew. & Pers.Norm × DonationsBehav1		−0.259		0.217
		(0.231)		(0.242)
				
Mon.Rew. & Soc.Norm × DonationsBehav1		$-0.489^{*}$		−0.013
		(0.224)		(0.235)
				
Constant	24.873${}^{***}$	−11.144	12.710${}^{***}$	0.612
	(7.341)	(11.232)	(7.970)	(13.040)
				

Observations	3,782	3,782	3,782	3,782
Log Likelihood	−9,599.269	−9,581.185	−9,599.839	−9,581.185

				
Reduced form regression results				

	*Dependent variable:*			
		DonationsBehav1		DonationsBehav1
		(1)		(2)

Donation value		11.643${}^{***}$		11.643${}^{***}$
		(0.144)		(0.144)
				
Constant		−0.216		−0.216
		(0.455)		(0.455)
				

Observations		3,782		3,782
R^2^		0.689		0.689
Adjusted R^2^		0.689		0.689

*Note:* Table shows the estimates of a tobit model regressing the donations in behaviour 2 on the treatment indicators in columns 1 and 3. Columns 2 and 4 report the 2SLS-regresssion results. The 2SLS uses an OLS regression in the reduced form, regressing *DonationsBehav1* on the donation amount assigned to breaking a code (bottom part of table), i.e. *DonationsBehav1* is instrumented by the assigned donation value in behaviour 1 for breaking a code. Second stage: Tobit regression. The dependent variable is given by survey points allocated to charity in behaviour 2. The independent variables comprise the treatment indicators and the interaction terms of the treatment indicators with the instrumented variable for donations in behaviour 1. Column 1 and 2 use the treatment indicator for the treatment *Prosocial only* and column 3 and 4 use the treatment indicator for the treatment *Mon.Rew.* as a base. Standard errors in parentheses. ${}^{+}$p < 0.1; ${}^{*}$p < 0.05; ${}^{**}$p < 0.01; ${}^{***}$p < 0.001.

**Table A.4:** Overall spillover effects on the extensive and intensive margin.

	*Dependent variable:*			
	Extensive margin		Intensive margin	
	*probit*		*OLS*	
	(1)	(2)	(3)	(4)
Personal Norm	0.132${}^{+}$	0.141${}^{*}$	1.224	1.994
	(0.069)	(0.071)	(3.327)	(3.275)
				
Social Norm	0.136${}^{*}$	0.158${}^{*}$	0.830	1.077
	(0.069)	(0.071)	(3.316)	(3.267)
				
Monetary Reward	0.014	0.054	$-9.924^{**}$	$-8.871^{**}$
	(0.068)	(0.070)	(3.374)	(3.356)
				
Mon.Rew. & Pers.Norm	0.113${}^{+}$	0.143${}^{*}$	−2.767	−2.716
	(0.068)	(0.071)	(3.309)	(3.281)
				
Mon.Rew. & Soc.Norm	0.173${}^{*}$	0.188${}^{**}$	−4.707	−4.693
	(0.068)	(0.071)	(3.286)	(3.277)
				
Constant	0.115${}^{**}$	$-0.925^{***}$	103.216${}^{***}$	55.899${}^{***}$
	(0.044)	(0.223)	(2.220)	(10.551)
				

Control variables		×		×
Observations	3,782	3,782	2,196	2,196
R^2^			0.007	0.050
Adjusted R^2^			0.004	0.040
Log Likelihood	−2,566.868	−2,391.021		

*Note:* Columns 1 and 2 report Probit model regression results. The dependent variable is given by whether donations had been greater than zero in behaviour 2, [0, 1]. Columns 3 and 4 show OLS estimates using the amount donated in behaviour 2 as a dependent variable. The sample is restricted to participants donating positive amounts to charity in behaviour 2. The treatment indicators are used as independent variables. Columns 2 and 4 additionally control for other covariates. The full regression table can be retrieved from the Supplementary Material, Table 3. Standard errors in parentheses. ${}^{+}$p < 0.1; ${}^{*}$p < 0.05; ${}^{**}$p < 0.01; ${}^{***}$p < 0.001.

**Table A.5:** Estimates of interventional spillover and persistence effect at the extensive and intensive margin.

	*Dependent variable:*			
	Extensive margin		Intensive margin	
	*probit*		*OLS*	
	(1)	(2)	(3)	(4)
DonationsBehav1	0.005${}^{***}$	0.005${}^{***}$	−0.014	−0.012
	(0.001)	(0.001)	(0.046)	(0.045)
				
Personal norm × DonationsBehav1	$-0.004^{**}$	$-0.004^{*}$	0.133${}^{+}$	0.140${}^{+}$
	(0.001)	(0.002)	(0.075)	(0.074)
				
Social norm × DonationsBehav1	$-0.002^{+}$	$-0.003^{*}$	0.029	0.038
	(0.001)	(0.001)	(0.070)	(0.069)
				
Monetary reward × DonationsBehav1	$-0.004^{**}$	$-0.004^{**}$	0.077	0.085
	(0.001)	(0.001)	(0.072)	(0.071)
				
Mon.Rew & Pers.Norm × DonationsBehav1	−0.002	−0.001	0.028	0.039
	(0.001)	(0.002)	(0.073)	(0.072)
				
Mon.Rew & Soc.Norm × DonationsBehav1	$-0.003^{*}$	$-0.004^{**}$	−0.033	−0.054
	(0.001)	(0.001)	(0.071)	(0.070)
				
Personal norm	0.321${}^{**}$	0.315${}^{**}$	−7.342	−7.036
	(0.113)	(0.116)	(5.812)	(5.730)
				
Social norm	0.234${}^{*}$	0.274${}^{*}$	−1.069	−1.397
	(0.109)	(0.113)	(5.661)	(5.568)
				
Monetary reward	0.226${}^{*}$	0.245${}^{*}$	$-14.862^{**}$	$-14.368^{*}$
	(0.108)	(0.111)	(5.674)	(5.602)
				
Mon.Rew & Pers.Norm	0.154	0.141	−4.699	−5.502
	(0.117)	(0.121)	(6.062)	(5.987)
				
Mon.Rew & Soc.Norm	0.296${}^{**}$	0.366${}^{**}$	−2.184	−0.744
	(0.113)	(0.117)	(5.799)	(5.742)
				
Constant	$-0.131^{*}$	$-1.180^{***}$	104.053${}^{***}$	56.837${}^{***}$
	(0.065)	(0.229)	(3.555)	(10.889)
				

Control variables		×		×
Observations	3,782	3,782	2,196	2,196
R^2^			0.010	0.054
Adjusted R^2^			0.005	0.041
Log Likelihood	−2,546.206	−2,370.420		

Reduced from regression				

	*Dependent variable:*			
	DonationsBehav1			
	(1)	(2)	(3)	(4)

Donation value	11.643${}^{***}$	11.643${}^{***}$	11.336${}^{***}$	11.336${}^{***}$
	(0.127)	(0.127)	(0.173)	(0.173)
				
Constant	−0.216	−0.216	0.308	0.308
	(0.885)	(0.885)	(1.240)	(1.240)
				

Observations	3,782	3,782	2,196	2,196
R^2^	0.689	0.689	0.661	0.661
Adjusted R^2^	0.689	0.689	0.661	0.661

*Note:* Table shows the second stages of the 2SLS-regresssion. OLS regression in the first stage, regressing *DonationsBehav1* on the donation amount assigned to breaking a code (see bottom part of table), i.e. *DonationsBehav1* is instrumented by the assigned donation value in behaviour 1 for breaking a code. Columns 1 and 2 report Probit model regression results from the second stage. The dependent variable is given by whether donations had been greater than zero in behaviour 2, [0, 1]. Columns 3 and 4 show OLS estimates using the amount donated in behaviour 2 as a dependent variable in the 2SLS second stage. The sample is restricted to participants donating positive amounts to charity in behaviour 2. The treatment indicators, the instrumented variable for amount donated in behaviour 1 and the interaction terms for the treatment indicators and the instrumented variable for amount donated in behaviour 1 are used as independent variables. Columns 2 and 4 additionally control for other covariates. Standard errors in parentheses. ${}^{+}$p < 0.1; ${}^{*}$p < 0.05; ${}^{**}$p < 0.01; ${}^{***}$p < 0.001.

**Table A.6:** Elicited norms and effort in behaviour 1.

	*Dependent variable:*			
	AmountCharity			
	(1)	(2)	(3)	(4)
Personal norm	$-2.291^{*}$	−1.511	0.730	1.018
	(1.082)	(0.945)	(0.987)	(0.819)
				
Social norm	0.215	0.245	−0.365	−0.165
	(1.056)	(0.944)	(1.093)	(0.936)
				
Mon.Rew. & Pers.Norm	−1.358	0.045	0.691	1.535${}^{+}$
	(0.965)	(0.835)	(0.975)	(0.796)
				
Mon.Rew. & Soc.Norm	1.244	1.422${}^{+}$	2.449${}^{*}$	2.248${}^{**}$
	(0.977)	(0.818)	(0.993)	(0.809)
				
Mon.Rew.	0.606	1.557${}^{+}$	1.462	1.854${}^{*}$
	(1.040)	(0.905)	(0.989)	(0.827)
				
personal norm Behav1	0.073	0.094		
	(0.077)	(0.068)		
				
Personal norm × personal norm Behav1	0.227${}^{*}$	0.170${}^{+}$		
	(0.104)	(0.091)		
				
Social norm × personal norm Behav1	−0.013	−0.008		
	(0.100)	(0.089)		
				
Mon.Rew._Personal norm × personal norm Behav1	0.140	0.023		
	(0.092)	(0.080)		
				
Mon.Rew. & Soc.Norm × personal norm Behav1	−0.080	−0.094		
	(0.096)	(0.082)		
				
Mon.Rew. × personal norm Behav1	−0.024	−0.109		
	(0.104)	(0.091)		
				
social norm Behav1			0.282${}^{**}$	0.231${}^{**}$
			(0.072)	(0.059)
				
Personal norm × social norm Behav1			−0.083	−0.086
			(0.091)	(0.076)
				
Social norm × social norm Behav1			0.037	0.030
			(0.103)	(0.086)
				
Mon.Rew._Personal norm × social norm Behav1			−0.058	−0.121
			(0.091)	(0.074)
				
Mon.Rew. & Soc.Norm × social norm Behav1			$-0.205^{*}$	$-0.179^{*}$
			(0.094)	(0.076)
				
Mon.Rew. × social norm Behav1			−0.106	$-0.133^{+}$
			(0.095)	(0.080)
				
Constant	10.671${}^{***}$	15.608${}^{***}$	8.585${}^{***}$	14.286${}^{***}$
	(0.774)	(0.965)	(0.746)	(0.948)
				

Control variables		×		×
Observations	3,036	3,036	3,036	3,036
R^2^	0.014	0.295	0.029	0.301
Adjusted R^2^	0.010	0.288	0.025	0.294

*Note:* OLS regression. The dependent variable is given by broken codes within the six minutes in behaviour 1. In column 1 and 2, the independent variables are given by the treatment indicators, the elicited personal norm parameter and the respective interaction terms. In columns 3 and 4, the independent variables are given by the treatment indicators, the elicited social norm parameter and the respective interaction terms. Columns 2 and 4 additionally controls for other covariates. The full estimation result can be retrieved from the Supplementary Material, Table 5. Robust standard errors in parentheses. ${}^{+}$p < 0.1; ${}^{*}$p < 0.05; ${}^{**}$p < 0.01; ${}^{***}$p < 0.001.

## Data Availability

The code for analysis is available from the corresponding author upon reasonable request. Experimental data is available at https://osf.io/zgqjs/ .

## Appendix A Additional tables and figures

See Fig. A.1 and Table A.1, Table A.2, Table A.3, Table A.4, Table A.5, Table A.6.

## Appendix B Supplementary data

MMC S1 Supplementary Material.

## Footnotes

1 Pro-environmental behaviour can be understood as a specific type of prosocial behaviour. Both behaviours benefit others, the society or the environment incurring private costs to an individual (Bénabou and Tirole, 2006, Brekke and Johansson-Stenman, 2008).

2 The term “norm” generally means what is commonly done, what is commonly perceived as acceptable, or what is commonly deemed the appropriate behaviour (Bicchieri, 2023). Here we specifically refer to the last conception of norm, that is, a common judgement of behaviour as appropriate.

3 Individuals may, in fact, derive intrinsic satisfaction from believing that their behaviour aligns with norms (Sripada and Stich, 2005).

4 One hundred survey points correspond to EUR 1 or PLN 2.6.

5 These questions enable us to obtain a sample representative of the country population according to these characteristics.

6 The food bank that receives the donation is based either in Italy (Banco Alimentare) or Poland (Banki Zywnosci), depending on the country of residence of the participant. The decision to provide participants with the choice to donate to a food bank was grounded in the motivation to address both social and environmental goals. Food banks prevent food waste by distributing to people in need edible food that for various reasons can no longer be sold. Additionally, this set-up allowed us to explore the crowding-out phenomenon, which is widely recognised as explaining the negative interactions of intrinsic motives and economic incentives with regard to both prosocial (Gneezy et al., 2011) and pro-environmental (Frey and Oberholzer-Gee, 1997) preferences.

7 In the analysis, we exploit this variation to differentiate between the *pure spillover effect* and the *interventional spillover effect*.

8 Social norms are collective perceptions regarding the appropriateness of an action. The standard of appropriateness depends on collective perceptions of correct behaviour, which, in turn, are conditional on others’ expectations of appropriate behaviour and can be enforced by external sanctions or their threat (Bicchieri, 2005, Bicchieri, 2010, Bašić and Verrina, 2023).

9 Personal norms are individual perceptions regarding the appropriateness of an action. The standard of appropriateness depends on individual perceptions of correct behaviour, are unconditional on social expectations and are subject to intrinsic sanctioning tied to the self-concept (Schwartz, 1973, Bicchieri, 2010, Bašić and Verrina, 2023).

10 These instructions may not explicitly articulate whether participants should consider what others consider appropriate for themselves, or what others consider appropriate for others, as they are primarily operationalised to capture the specific normative focus of our study, namely the injunctive aspect of social norms (i.e. the collective perception of appropriate behaviour) and personal norms (i.e. the personal perception of appropriate behaviour).

11 See Supplementary material, Section 3.

12 While we cannot completely rule out the possibility that the filler task has an effect on behaviour 2, we note that the filler task consists of questions unrelated to the elicitation of behaviour 2. Therefore, we do not expect such an effect to occur.

13 According to standard economic theory, the higher the endowment available for disposal, the lower the marginal utility of this money and, theoretically, the greater the participants’ likelihood to donate. However, the actual impact in the experiment may be rather small. Instead, the magnitude of the income effect on donation decisions may vary, considering that individuals may differ in their mental accounting (Thaler, 1999). In other words, participants may differ in how they treat the monetary remuneration obtained from performing behaviour 1 separately from the decision to donate (behaviour 2). To address this potentially different income effect, we ensure that the distribution of disposable endowment among participants is similar across the six treatments.

14 In particular, every participant who breaks fewer than seven codes in behaviour 1 receive “70points−codes solved×10points” for answering the questions in the filler task. In the respective treatments, this creates a lower bound to the distribution of earnings at the stage of behaviour 2.

15 Fig. A.1 in Appendix A shows the distributions of earnings across treatments.

16 The preregistered hypotheses provided initial expectations about spillover effects. However, because we characterised spillover effects more comprehensively after preregistration, the predictions presented here reflect this more comprehensive characterisation of spillover effects. Because our focus shifted to examining these broader spillover effects (i.e. on the second prosocial behaviour) from policy mixes, we do not include the hypotheses originally preregistered (hypothesis 1a, hypothesis 2a) regarding the first prosocial behaviour.

17 We treat personal and social norm compliance dimensions separately to better capture the various channels through which norm nudges can be effective. This approach aligns with empirical evidence showing that personal and social norm dimensions can have different impacts on individual behaviour (Capraro and Rand, 2018, Catola et al., 2021, Bašić and Verrina, 2023).

18 Here, we use the term “social norm” in a generic sense, referring specifically to injunctive norms that prescribe socially perceived behaviours (Bicchieri, 2005). This focus on the injunctive aspect allows us to describe the alignment between personal and collective perceptions of appropriate behaviour.

19 Acknowledging the potential overlap between “self-view” and “personal norm compliance”, it can be argued that an individual’s self-perception improves when they behave in line with their personal standards of appropriateness. However, we treat these as distinct. This approach aligns with Festré (2010). In this context, the “self-view” component, which considers past behaviour explicitly, enables us to account for a potential licensing effect that would be overlooked if only the personal norm compliance component were considered.

20 Hypotheses 1b and 2b in the preregistration examine the influence of monetary rewards alone and norm nudges alone on the likelihood of engaging in prosocial behaviour later on. Given our primary focus on spillover effects from policy mixes, we opt to include these hypotheses on single interventions only in the main text.

21 Predictions 2a, 2b, 2c, 3a, 3b and 3c represent an evolution of hypothesis 3 from our preregistration. While hypothesis 3 initially posited the effectiveness of monetary rewards and moral nudges on prosocial behaviour, our refined predictions now emphasise the impacts of policy mixes involving personal norm nudges (predictions 2a, 2b and 2c) and social norm nudges (predictions 3a, 3b and 3c) based on our evolved characterisation of spillover effects (overall spillover effect, consisting of interventional and persistence effects).

22 Ipsos was responsible for questionnaire optimisation, scripting and translations of the experiment, the pilot trial ahead of main fieldwork, participant recruitment from Ipsos’s Online Access Panels, data collection, data processing and data delivery.

23 The experiment was conducted in these two countries representative of southern and eastern regions of the EU, to comply with the requirement of our funding institution that all studies should aim to include representation from at least two of the main EU regions (east, north, south, and west). The decision was also influenced by the availability in these two countries of a pool from which a representative sample to be surveyed could be selected. Data pooling was performed as no significant differences in between-treatment effects were observed between the countries in key variables (see Supplementary material, Section 1, Table 9).

24 As mentioned in the preregistration, we aimed for and achieved at least 589 observations per treatment to ensure sufficient power for the within-country analysis and the sub-sample analysis. We also managed to achieve the planned higher number of observations (N = 800) required for the ‘Prosocial’ treatment, necessary to determine whether we observe a spillover effect at all. Note that these observations only include participants who completed the experiment, as mentioned in the preregistration.

25 Our preregistration envisaged the inclusion of control variables, but it did not specify the variables to be included.

26 Note that the marginal effects reported are in Credits. One point translates to EUR 0.01.

27 The *overall spillover effect* and its differences across treatments are obtained from the parameters $\sum_{k=1}\beta_{j}\cdot X_{i,j}$ and $\sum_{k=1}\beta_{k}x_{i,1}\cdot X_{i,k}$ in the regression model provided in Supplementary material, Section 2.3.

28 There is no evidence that *overall spillover effects* of the policy mixes are smaller than those of the *personal norm nudge* (monetary reward x personal norm nudge, p = 0.3298; monetary reward x social norm nudge, p = 0.3954, see Table A.3, column 1 in Appendix A) and the *social norm nudge* treatment (monetary reward x personal norm nudge, p = 0.4116; monetary reward x social norm nudge, p = 0.4888, see Table A.3, column 1 in Appendix A).

29 The null effect is further supported by the data as we find no significant *Interventional spillover effect* on the extensive and intensive margin of donations (extensive margin: p = 0.3294, intensive margin: p = 0.7151, Table A.5 - Columns 2,4 in Appendix).
