# Supplementary material for: The more the better? Synergies of prosocial interventions and effects on behavioural spillovers
Source: J Environ Econ Manage. 2024 Nov;128:None. doi: 10.1016/j.jeem.2024.103061 (PMC11639204; doi:10.1016/j.jeem.2024.103061)
Supplement: MMC S1 — Supplementary Material. [file mmc1.pdf]

Supplementary Material

The more the better?
Synergies of prosocial interventions and effects on behavioural spillovers

This version: August, 2024

1 Additional Tables **1**

2 Additional analysis **19**

2.1 Identification strategy 19

2.2 Utility framework 22

2.3 Estimation model of the overall spillover effect 23

3 Script of Experiment **24**

1 Additional Tables

Table 1: Donations in behaviour 2 by treatments (donors in behaviour 1 only)

	Dependent variable:
	DonationsBehav2
pnorm	8.348
	(12.232)
snorm	7.910
	(12.152)
monrew	-27.917^{*}
	(11.796)
monrew_pnorm	-5.146
	(11.815)
monrew_snorm	-1.442
	(11.525)
Constant	50.941^{***}
	(8.270)
Subsample	DonationsBehav1 0
Observations	3,036
Log Likelihood	$-7,977.079$
Wald Test	12.021^{*} (df = 5)
Note:	*p<0.1; **p<0.05; ***p<0.01

Table 2: Performance in the decoding task by treatment

	Dependent variable:		
	CodesSolved		
	(1)	(2)	(3)
PersNorm	0.118	0.652	0.316
	(0.263)	(0.528)	(0.226)
SocNorm	0.189	1.385^{***}	0.310
	(0.255)	(0.519)	(0.217)
Mon.Rew.	0.315	1.215^{**}	0.454^{**}
	(0.250)	(0.481)	(0.211)
Mon.Rew. x PersNorm	0.272	1.225^{**}	0.475^{**}
	(0.255)	(0.492)	(0.217)
Mon.Rew. x SocNorm	0.478^{*}	0.644	0.522^{**}
	(0.258)	(0.512)	(0.218)
exhaust			-0.721^{***}
			(0.071)
tablet			-0.972^{**}
			(0.422)
smartphone			-2.189^{***}
			(0.142)
pastdon			-0.158^{***}
			(0.048)
Amount2			−0.007
			(0.016)
education			0.115^{***}
			(0.027)
age			-0.122^{***}
			(0.004)
female			0.569^{***}
			(0.139)
risk			−0.039
			(0.037)
prosocialnorm			0.030
			(0.080)
trust			-0.228^{**}
			(0.098)
timepref			0.186^{**}
			(0.080)
cr			0.381^{***}
			(0.045)
agency			0.041^{***}
			(0.014)

altruism			-0.182^{***}
			(0.051)
enbeh			0.027
			(0.025)
language			0.040
			(0.607)
vote_right			0.535^{***}
			(0.183)
speeder			0.927^{***}
			(0.256)
cont			-0.699^{***}
			(0.079)
Constant	11.343^{***}	11.000^{***}	16.730^{***}
	(0.166)	(0.316)	(0.684)
Observations	3,782	938	3,782
Log Likelihood	−11,167.650	−2,766.152	−10,509.280
Wald Test	4.065 (df = 5)	11.278^{**} (df = 5)	$1,569.182^{***}$ (df = 25)

Note: OLS regression. The dependent variable is given by broken codes within the six minutes in behaviour 1. In column 1 and 2, we use only the treatment indicators as independent variables. Column 2 repeats the column 1's estimation on a restricted sample of participants who have a below median value in the altruism variable inquired in the post-questionnaire. Column 3 additionally controls for other covariates. Robust standard errors in parentheses. $^{+}$p<0.1; *p<0.05; **p<0.01; ***p<0.001

Table 3: Interventions and spillover effects

	Dependent variable:							
	DonationsBehav2							
	(1)	(2)	(3)	(4)	(5)	(6)	(7)	(8)
DonationsBehave1	0.665***	0.602***			0.189	0.235		
	(0.147)	(0.139)			(0.164)	(0.155)		
pnorm $*$DonationsBehave1	−0.366	−0.276			0.110	0.091		
	(0.248)	(0.232)			(0.258)	(0.242)		
snorm $*$DonationsBehave1	−0.295	−0.275			0.181	0.093		
	(0.226)	(0.212)			(0.237)	(0.223)		
monrew $*$DonationsBehave1	−0.476**	−0.367*						
	(0.220)	(0.208)						
prosocial $*$DonationsBehave1					0.476**	0.367*		
					(0.220)	(0.208)		
monrew_pnorm $*$DonationsBehave1	−0.259	−0.133			0.217	0.235		
	(0.231)	(0.217)			(0.242)	(0.227)		
monrew_snorm $*$DonationsBehave1	−0.489**	−0.561***			−0.013	−0.194		
	(0.224)	(0.211)			(0.235)	(0.222)		
pnorm	42.206**	38.226**	25.530**	22.927**	30.450	25.841	31.887***	31.887***
	(18.959)	(17.746)	(11.330)	(10.534)	(20.072)	(18.957)	(11.156)	(11.156)
snorm	34.697*	35.789**	23.595**	20.520*	22.941	23.403	29.479***	29.479***
	(18.158)	(16.848)	(11.184)	(10.510)	(19.318)	(18.104)	(11.086)	(11.086)
monrew	11.756	12.385	−12.163	−8.960				
	(17.067)	(16.248)	(10.741)	(10.248)				
prosocial					−11.756	−12.385	8.960	8.960
					(17.067)	(16.248)	(10.248)	(10.248)
monrew_pnorm	20.156	14.021	13.937	9.010	8.401	1.636	17.969*	17.969*
	(19.240)	(18.246)	(10.956)	(10.443)	(20.335)	(19.297)	(10.878)	(10.878)
monrew_snorm	39.326**	44.442**	15.615	8.749	27.570	32.057*	17.709*	17.709*
	(18.333)	(17.454)	(10.697)	(10.278)	(19.486)	(18.523)	(10.647)	(10.647)
AmountEarned		0.053		0.050		0.053	0.050	0.050
		(0.094)		(0.094)		(0.094)	(0.094)	(0.094)
exhaust		0.903		0.841		0.903	0.841	0.841
		(3.035)		(3.034)		(3.035)	(3.034)	(3.034)

tablet	−22.835	−24.351	−22.835	−24.351	−24.351
	(24.297)	(24.302)	(24.297)	(24.302)	(24.302)
smartphone	−52.495***	−52.294***	−52.495***	−52.294***	−52.294***
	(7.143)	(7.137)	(7.143)	(7.137)	(7.137)
pastdon	13.419***	13.337***	13.419***	13.337***	13.337***
	(2.303)	(2.303)	(2.303)	(2.303)	(2.303)
Amount2		4.080***		4.080***	4.080***
		(0.772)		(0.772)	(0.772)
education	−0.507	−0.460	−0.507	−0.460	−0.460
	(1.309)	(1.309)	(1.309)	(1.309)	(1.309)
age	1.397***	1.386***	1.397***	1.386***	1.386***
	(0.219)	(0.219)	(0.219)	(0.219)	(0.219)
female	−6.375	−6.470	−6.375	−6.470	−6.470
	(6.457)	(6.464)	(6.457)	(6.464)	(6.464)
risk	0.176	0.146	0.176	0.146	0.146
	(1.721)	(1.721)	(1.721)	(1.721)	(1.721)
prosocialnorm	10.891***	10.953***	10.891***	10.953***	10.953***
	(3.843)	(3.842)	(3.843)	(3.842)	(3.842)
trust	27.140***	27.342***	27.140***	27.342***	27.342***
	(4.630)	(4.638)	(4.630)	(4.638)	(4.638)
timepref	18.037***	18.007***	18.037***	18.007***	18.007***
	(3.838)	(3.841)	(3.838)	(3.841)	(3.841)
cr	2.538	2.753	2.538	2.753	2.753
	(2.111)	(2.110)	(2.111)	(2.110)	(2.110)
agency	−0.809	−0.788	−0.809	−0.788	−0.788
	(0.705)	(0.705)	(0.705)	(0.705)	(0.705)
altruism	10.656***	10.552***	10.656***	10.552***	10.552***
	(2.489)	(2.491)	(2.489)	(2.491)	(2.491)
enbeh	−2.677**	−2.704**	−2.677**	−2.704**	−2.704**
	(1.171)	(1.172)	(1.171)	(1.172)	(1.172)
language	36.356	36.531	36.356	36.531	36.531
	(26.836)	(26.870)	(26.836)	(26.870)	(26.870)
vote_right	−16.383*	−16.116*	−16.383*	−16.116*	−16.116*
	(8.380)	(8.392)	(8.380)	(8.392)	(8.392)

	(1)	(2)	(3)	(4)	(5)	(6)	(7)	(8)
speeder		1.543		1.577		1.543	1.577	1.577
		(11.858)		(11.883)		(11.858)	(11.883)	(11.883)
cont		0.259		0.629		0.259	0.629	0.629
		(3.800)		(3.802)		(3.800)	(3.802)	(3.802)
Constant	−11.144	−187.840***	24.873***	−174.934***	0.612	−175.454***	−183.894***	−183.894***
	(11.232)	(34.948)	(7.341)	(34.333)	(13.040)	(36.002)	(35.093)	(35.093)
Observations	3,782	3,782	3,782	3,782	3,782	3,782	3,782	3,782
Log Likelihood	−9,581.185	−9,376.790	−9,599.269	−9,380.839	−9,581.185	−9,376.790	−9,380.839	−9,380.839

Note: Table shows the second stage of the 2SLS-regresssion in column 1,2,5, and 6. OLS regression in the first stage, regressing *DonationsBehave1* on the donation amount assigned to breaking a code (see Supplementary Material, Table 2), i.e. *DonationsBehave1* is instrumented by the assigned donation value in behaviour 1 for breaking a code. Second stage: Tobit regression. The dependent variable is given by survey points allocated to charity in behaviour 2. The independent variables comprise the treatment indicators and the interaction terms of the treatment indicators with the instrumented variable for donations in behaviour 1. Column 1 and 2 use the treatment indicator for the treatment *Prosocial only* and column 5 and 6 use the treatment indicator for the treatment *Mon.Rew.* as a base. Columns 3,4,7, and 8 show OLS estimates using the amount donated in behaviour 2 as a dependent variable and the treatment indicators as independent variables. Columns 2, 4, 6, and 8 additionally control for other covariates. The full regression table can be retrieved from the Supplementary Material, Table 3. Standard errors in parentheses. $^{+}$p<0.1; *p<0.05; **p<0.01; ***p<0.001

Table 4: Overall spillover effects on the extensive and intensive margin

	Dependent variable:			
	extensive margin		intensive margin	
	probit		*OLS*	
	(1)	(2)	(3)	(4)
PerSocNorm	0.132*	0.141**	1.224	1.994
	(0.069)	(0.071)	(3.327)	(3.275)
SocNorm	0.136**	0.158**	0.830	1.077
	(0.069)	(0.071)	(3.316)	(3.267)
Mon.Rew.	0.014	0.054	−9.924***	−8.871***
	(0.068)	(0.070)	(3.374)	(3.356)
Mon.Rew. x PerSocNorm	0.113*	0.143**	−2.767	−2.716
	(0.068)	(0.071)	(3.309)	(3.281)
Mon.Rew. x SocNorm	0.173**	0.188***	−4.707	−4.693
	(0.068)	(0.071)	(3.286)	(3.277)
AmountEarned		0.0001		0.037
		(0.001)		(0.030)
exhaust		0.020		−1.371
		(0.021)		(0.977)
tablet		−0.292*		5.995
		(0.151)		(7.178)

smartphone		−0.350***		−4.031*
		(0.048)		(2.261)
pastdon		0.101***		−0.054
		(0.015)		(0.740)
education		−0.005		−0.124
		(0.009)		(0.409)
age		0.006***		0.399***
		(0.002)		(0.070)
female		−0.038		1.090
		(0.045)		(2.048)
risk		−0.010		1.171**
		(0.012)		(0.539)
Prosocialnorm		0.080***		0.497
		(0.025)		(1.210)
trust		0.175***		3.977***
		(0.031)		(1.449)
timepref		0.116***		1.514
		(0.026)		(1.231)
cr		−0.0001		1.274*
		(0.015)		(0.669)
agency		−0.010**		0.197
		(0.005)		(0.220)
altruism		0.067***		0.516
		(0.016)		(0.768)
language		0.179		0.487
		(0.189)		(8.697)
vote_right		−0.063		−6.244**
		(0.057)		(2.692)
speeder		0.021		−1.506
		(0.078)		(3.854)
cont		−0.036		1.387
		(0.026)		(1.209)
Constant	0.115***	−0.925***	103.216***	55.899***
	(0.044)	(0.223)	(2.220)	(10.551)
Observations	3,782	3,782	2,196	2,196
R^2			0.007	0.050
Adjusted R^2			0.004	0.040
Log Likelihood	−2,566.868	−2,391.021		
Akaike Inf. Crit.	5,145.736	4,832.041		
Residual Std. Error			46.356 (df = 2190)	45.521 (df = 2171)
F Statistic			2.946** (df = 5; 2190)	4.805*** (df = 24; 2171)

Note: Columns 1 and 2 report Probit model regression results. The dependent variable is given by whether donations had been greater than zero in behaviour 2, [0,1]. Columns 3 and 4 show OLS estimates using the amount donated in behaviour 2 as a dependent variable. The sample is restricted to participants donating positive amounts to charity in behaviour 2. The treatment indicators are used as independent variables. Columns 2 and 4 additionally control for other covariates. Standard errors in parentheses. $^{+}$p<0.1; *p<0.05; **p<0.01; ***p<0.001

Table 5: Estimates of internventional spillover and persistence effect at the extensive and intensive margin

	Dependent variable:			
	Extensive margin		Intensive margin	
	probit		*OLS*	
	(1)	(2)	(3)	(4)
DonationsBehave1	0.005***	0.005***	−0.014	−0.012
	(0.001)	(0.001)	(0.046)	(0.045)
PerSocNorm ∗DonationsBehave1	−0.004***	−0.004**	0.133*	0.140*
	(0.001)	(0.002)	(0.075)	(0.074)
SocNorm ∗DonationsBehave1	−0.002*	−0.003**	0.029	0.038
	(0.001)	(0.001)	(0.070)	(0.069)
Mon.Rew. ∗DonationsBehave1	−0.004***	−0.004***	0.077	0.085
	(0.001)	(0.001)	(0.072)	(0.071)
Mon.Rew._PerSocNorm ∗DonationsBehave1	−0.002	−0.001	0.028	0.039
	(0.001)	(0.002)	(0.073)	(0.072)
Mon.Rew. x SocNorm ∗DonationsBehave1	−0.003**	−0.004***	−0.033	−0.054
	(0.001)	(0.001)	(0.071)	(0.070)
PerSocNorm	0.321***	0.315***	−7.342	−7.036
	(0.113)	(0.116)	(5.812)	(5.730)
SocNorm	0.234**	0.274**	−1.069	−1.397
	(0.109)	(0.113)	(5.661)	(5.568)
Mon.Rew.	0.226**	0.245**	−14.862***	−14.368**
	(0.108)	(0.111)	(5.674)	(5.602)
Mon.Rew. x PerSocNorm	0.154	0.141	−4.699	−5.502
	(0.117)	(0.121)	(6.062)	(5.987)
Mon.Rew. x SocNorm	0.296***	0.366***	−2.184	−0.744
	(0.113)	(0.117)	(5.799)	(5.742)
AmountEarned		0.0001		0.037
		(0.001)		(0.030)
exhaust		0.019		−1.448
		(0.021)		(0.977)
tablet		−0.272*		5.917
		(0.152)		(7.187)
smartphone		−0.356***		−4.172*
		(0.048)		(2.263)

pastdon		0.102***		−0.086
		(0.015)		(0.741)
education		−0.005		−0.151
		(0.009)		(0.409)
age		0.007***		0.405***
		(0.002)		(0.070)
female		−0.039		1.050
		(0.045)		(2.048)
risk		−0.012		1.148**
		(0.012)		(0.540)
Prosocialnorm		0.074***		0.463
		(0.025)		(1.212)
trust		0.170***		4.030***
		(0.032)		(1.449)
timepref		0.125***		1.502
		(0.026)		(1.232)
cr		0.002		1.237*
		(0.015)		(0.671)
agency		−0.010**		0.200
		(0.005)		(0.220)
altruism		0.068***		0.644
		(0.017)		(0.770)
language		0.204		0.198
		(0.190)		(8.700)
vote_right		−0.067		−6.212**
		(0.057)		(2.693)
speeder		0.024		−1.627
		(0.078)		(3.853)
cont		−0.023		1.468
		(0.026)		(1.216)
Constant	−0.131**	−1.180***	104.053***	56.837***
	(0.065)	(0.229)	(3.555)	(10.889)
Observations	3,782	3,782	2,196	2,196
R^2			0.010	0.054
Adjusted R^2			0.005	0.041
Log Likelihood	−2,546.206	−2,370.420		

Note: Table shows the second stages of the 2SLS-regresssion. OLS regression in the first stage, regressing *Donations-Behave1* on the donation amount assigned to breaking a code (see Supplementary Material, Table 2), i.e. *Donations-Behave1* is instrumented by the assigned donation value in behaviour 1 for breaking a code. Columns 1 and 2 report Probit model regression results from the second stage. The dependent variable is given by whether donations had been greater than zero in behaviour 2, [0,1]. Columns 3 and 4 show OLS estimates using the amount donated in behaviour 2 as a dependent variable in the 2SLS second stage. The sample is restricted to participants donating positive amounts to charity in behaviour 2. The treatment indicators, the instrumented variable for amount donated in behaviour 1 and the interaction terms for the treatment indicators and the instrumented variable for amount donated in behaviour 1 are used as independent variables. Columns 2 and 4 additionally control for other covariates. The full regression table can be retrieved from the Supplementary Material, Table 4. Standard errors in parentheses. $^{+}$p<0.1; *p<0.05; **p<0.01; ***p<0.001

Table 6: Elicited norms and effort in behaviour 1

	Dependent variable:			
	CodesSolved			
	Tobit			
	(1)	(2)	(3)	(4)
DonationsBehave1	1.115	0.934	−0.003	−0.425
	(0.738)	(0.717)	(0.005)	(0.668)
PersNorm ∗DonationsBehave1	−0.881	−0.847	−0.002	−0.004
	(1.133)	(1.072)	(0.006)	(0.967)
SocNorm ∗DonationsBehave1	-1.961^{*}	-2.233^{**}	0.002	−0.509
	(1.058)	(1.011)	(0.007)	(1.011)
Mon.Rew. ∗DonationsBehave1	−0.808	−0.524	0.004	0.698
	(0.993)	(0.941)	(0.006)	(0.883)
Mon.Rew. x PersNorm ∗DonationsBehave1	-2.684^{***}	-2.305^{**}	−0.004	−0.626
	(1.011)	(0.979)	(0.006)	(0.934)
Mon.Rew. x SocNorm ∗DonationsBehave1	−0.788	−0.650	0.010	0.721
	(0.997)	(0.964)	(0.007)	(0.901)
PersNorm	62.646	57.526	0.193	30.122
	(97.009)	(91.610)	(0.564)	(83.317)
SocNorm	165.470^{*}	213.653^{**}	−0.354	31.453
	(97.737)	(93.088)	(0.619)	(92.624)
Mon.Rew. x PersNorm	231.909^{**}	210.855^{**}	0.291	56.927
	(92.522)	(90.150)	(0.587)	(86.209)
Mon.Rew. x SocNorm	13.331	2.345	−0.903	−75.663
	(93.333)	(90.104)	(0.596)	(84.630)
Mon.Rew.	−27.591	−42.853	−0.731	−113.354
	(84.349)	(80.067)	(0.540)	(76.178)
elicited pers. norm	5.902	5.278		
	(6.524)	(6.262)		
PersNorm ∗elicited pers. norm	−3.893	−3.766		
	(9.409)	(8.920)		

SocNorm ∗elicited pers. norm	−14.545	−18.402**	
	(9.162)	(8.731)	
Mon.Rew. x PersNorm ∗elicited pers. norm	−23.012***	−21.571**	
	(8.655)	(8.393)	
Mon.Rew. x SocNorm ∗elicited pers. norm	1.214	3.782	
	(8.919)	(8.591)	
Mon.Rew. ∗elicited pers. norm	3.193	5.223	
	(8.433)	(7.962)	
PersNorm ∗elicited pers. norm ∗DonationsBehave1	0.070	0.074	
	(0.108)	(0.103)	
SocNorm ∗elicited pers. norm ∗DonationsBehave1	0.184*	0.205**	
	(0.101)	(0.096)	
Mon.Rew. x PersNorm ∗elicited pers. norm ∗DonationsBehave1	0.262***	0.235**	
	(0.095)	(0.091)	
Mon.Rew. x SocNorm ∗elicited pers. norm ∗DonationsBehave1	0.046	0.015	
	(0.097)	(0.093)	
Mon.Rew. ∗elicited pers. norm ∗DonationsBehave1	0.046	0.022	
	(0.099)	(0.093)	
elicited pers. norm ∗DonationsBehave1	−0.091	−0.075	
	(0.074)	(0.071)	
AmountEarned		0.053	0.031
		(0.102)	(0.103)
exhaust		0.125	0.116
		(3.250)	(3.272)
tablet		−35.944	−38.159
		(25.252)	(25.313)
smartphone		−49.728***	−49.762***
		(7.646)	(7.669)
pastdon		13.100***	12.913***
		(2.460)	(2.462)
education		−0.119	−0.163
		(1.391)	(1.397)
age		1.327***	1.320***
		(0.234)	(0.234)
female		−5.393	−4.990
		(6.908)	(6.922)
risk		1.071	1.187
		(1.849)	(1.855)
prosocialnorm		8.558**	8.908**
		(4.143)	(4.130)
trust		25.670***	25.293***
		(4.895)	(4.892)
timepref		15.076***	14.610***

		(4.099)		(4.110)
cr		1.935		2.048
		(2.262)		(2.272)
agency		−1.002		−1.098
		(0.752)		(0.749)
altruism		7.237***		7.219***
		(2.611)		(2.616)
language		30.687		24.216
		(31.139)		(30.907)
vote_right		−16.131*		−15.962*
		(8.994)		(9.047)
speeder		5.447		5.551
		(13.150)		(13.169)
cont		5.494		5.322
		(4.068)		(4.080)
elicited soc. norm			−0.043	−4.101
			(0.040)	(5.684)
PersNorm *elicited soc. norm			0.0004	−0.485
			(0.052)	(7.705)
SocNorm *elicited soc. norm			0.044	−0.917
			(0.058)	(8.656)
Mon.Rew. x PersNorm *elicited soc. norm			−0.037	−6.735
			(0.054)	(7.972)
Mon.Rew. x SocNorm *elicited soc. norm			0.119**	11.641
			(0.056)	(7.800)
Mon.Rew. *elicited soc. norm			0.085	11.967
			(0.052)	(7.293)
PersNorm *elicited soc. norm *DonationsBehave1			−0.00005	−0.016
			(0.001)	(0.088)
SocNorm *elicited soc. norm *DonationsBehave1			−0.0002	0.038
			(0.001)	(0.096)
Mon.Rew. x PersNorm *elicited soc. norm *DonationsBehave1			0.0005	0.072
			(0.001)	(0.086)
Mon.Rew. x SocNorm *elicited soc. norm *DonationsBehave1			−0.001**	−0.124
			(0.001)	(0.083)
Mon.Rew. *elicited soc. norm *DonationsBehave1			−0.001	−0.098
			(0.001)	(0.083)
elicited soc. norm *DonationsBehave1			0.0005	0.064
			(0.0005)	(0.064)
Constant	−25.015	−213.363***	0.625	−116.331*
	(65.409)	(71.667)	(0.416)	(68.238)
Observations	3,036	3,036	3,036	3,036
Log Likelihood	−7,964.592	−7,812.182	−2,009.985	−7,816.037

Note: OLS regression. The dependent variable is given by broken codes within the six minutes in behaviour 1. In column 1 and 2, the independent variables are given by the treatment indicators, the elicited personal norm parameter and the respective interaction terms. In column 3 and 4, the independent variables are given by the treatment indicators, the elicited social norm parameter and the respective interaction terms. Columns 2 and 4 additionally controls for other covariates. Robust standard errors in parentheses. $^{+}$p<0.1; *p<0.05; **p<0.01; ***p<0.001

Table 7: Norms and spillover effects on the extensive margin of donations in behaviour 2

	Dependent variable:			
	DonationsBehav2			
	(1)	(2)	(3)	(4)
DonationsBehave1	0.006	0.005	−0.003	−0.003
	(0.005)	(0.005)	(0.005)	(0.005)
Personal norm x DonationsBehave1	−0.009	−0.009	−0.002	−0.001
	(0.007)	(0.007)	(0.006)	(0.007)
Social norm x DonationsBehave1	−0.009	−0.011	0.002	−0.002
	(0.007)	(0.007)	(0.007)	(0.007)
Mon.Rew. x DonationsBehave1	−0.006	−0.004	0.004	0.003
	(0.006)	(0.007)	(0.006)	(0.006)
Mon.Rew. & Pers.Norm x DonationsBehave1	-0.015^{*}	-0.012^{+}	−0.004	−0.002
	(0.007)	(0.007)	(0.006)	(0.007)
Mon.Rew. & Soc.Norm x DonationsBehave1	0.002	0.004	0.010	0.010
	(0.007)	(0.007)	(0.007)	(0.007)
Personal norm	0.428	0.439	0.193	0.213
	(0.602)	(0.618)	(0.564)	(0.580)
Social norm	0.676	1.052	−0.354	0.021
	(0.638)	(0.657)	(0.619)	(0.640)
Mon.Rew. & Pers.Norm	1.235^{*}	1.152^{+}	0.291	0.248
	(0.609)	(0.631)	(0.587)	(0.605)
Mon.Rew. & Soc.Norm	−0.399	−0.555	−0.903	−0.818
	(0.615)	(0.632)	(0.596)	(0.615)
Mon.Rew.	−0.190	−0.266	−0.731	−0.632
	(0.560)	(0.576)	(0.540)	(0.556)
elicited pers. norm	0.022	0.017		
	(0.042)	(0.044)		
Personal norm x elicited pers. norm	−0.027	−0.029		
	(0.058)	(0.060)		
Social norm x elicited pers. norm	−0.057	−0.083		
	(0.060)	(0.061)		
Mon.Rew. & Pers.Norm x elicited pers. norm	-0.127^{*}	-0.122^{*}		
	(0.056)	(0.058)		
Mon.Rew. & Soc.Norm x elicited pers. norm	0.070	0.099		
	(0.059)	(0.061)		

Mon.Rew. x elicited pers. norm	0.033	0.045		
	(0.057)	(0.058)		
Personal norm x elicited pers. norm x DonationsBehave1	0.001	0.001		
	(0.001)	(0.001)		
Social norm x elicited pers. norm x DonationsBehave1	0.001	0.001		
	(0.001)	(0.001)		
Mon.Rew. & Pers.Norm x elicited pers. norm x DonationsBehave1	0.001*	0.001*		
	(0.001)	(0.001)		
Mon.Rew. & Soc.Norm x elicited pers. norm x DonationsBehave1	−0.0005	−0.001		
	(0.001)	(0.001)		
Mon.Rew. x elicited pers. norm x DonationsBehave1	0.0003	0.0001		
	(0.001)	(0.001)		
elicited pers. norm x DonationsBehave1	−0.0005	−0.0003		
	(0.0005)	(0.001)		
elicited soc. norm			−0.043	−0.035
			(0.040)	(0.041)
Personal norm x elicited soc. norm			0.0004	−0.003
			(0.052)	(0.053)
Social norm x elicited soc. norm			0.044	0.015
			(0.058)	(0.060)
Mon.Rew. & Pers.Norm x elicited soc. norm			−0.037	−0.037
			(0.054)	(0.056)
Mon.Rew. & Soc.Norm x elicited soc. norm			0.119*	0.123*
			(0.056)	(0.057)
Mon.Rew. x elicited soc. norm			0.085	0.078
			(0.052)	(0.054)
Personal norm x elicited soc. norm x DonationsBehave1			−0.00005	−0.0001
			(0.001)	(0.001)
Social norm x elicited soc. norm x DonationsBehave1			−0.0002	−0.00000
			(0.001)	(0.001)
Mon.Rew. & Pers.Norm x elicited soc. norm x DonationsBehave1			0.0005	0.0003
			(0.001)	(0.001)
Mon.Rew. & Soc.Norm x elicited soc. norm x DonationsBehave1			−0.001*	−0.001*
			(0.001)	(0.001)
Mon.Rew. x elicited soc. norm x DonationsBehave1			−0.001	−0.001
			(0.001)	(0.001)
elicited soc. norm x DonationsBehave1			0.0005	0.0005
			(0.0005)	(0.0005)
Constant	−0.017	−1.074*	0.625	−0.516
	(0.429)	(0.501)	(0.416)	(0.493)
Control variables		X		X
Observations	3,036	3,036	3,036	3,036
Log Likelihood	−2,008.199	−1,879.095	−2,009.985	−1,883.732

Akaike Inf. Crit.	4,064.397	3,844.190	4,067.970	3,853.465

Note: Table shows an indicator for donations in behaviour 2 being greater than 0 (effects on the extensive margin of donations) as a dependent variable regressed on treatment effects and interactions with the elicited norms on the extensive margin using a Probit mode. The estimates present the 2SLS-regresssion. OLS regression in the first stage, regressing *DonationsBehav1* on the donation amount assigned to breaking a code (see bottom part of Table A5 - Column 1 and 2 in Appendix), i.e. *DonationsBehav1* is instrumented by the assigned donation value in behaviour 1 for breaking a code. The treatment indicators, the instrumented variable for amount donated in behaviour 1 and the interaction terms for the treatment indicators and the instrumented variable for amount donated in behaviour 1 are used as independent variables. Robust standard errors in parenthesis, $^{+}$p<0.1; *p<0.05; ***p<0.01

Table 8: Norms and spillover effects on the intensive margin of donations in behaviour 2

	Dependent variable:			
	DonationsBehav2			
	OLS			
	(1)	(2)	(3)	(4)
	Dependent variable:			
	DonationsBehav2			
	(1)	(2)	(3)	(4)
DonationsBehav1	−0.022	0.041	−0.043	0.001
	(0.165)	(0.163)	(0.169)	(0.167)
Personal norm x DonationsBehav1	0.379	0.305	0.126	0.110
	(0.269)	(0.265)	(0.246)	(0.243)
Social norm x DonationsBehav1	−0.329	−0.388	−0.231	−0.317
	(0.255)	(0.252)	(0.265)	(0.262)
Monetary reward x DonationsBehav1	0.181	0.149	0.133	0.093
	(0.254)	(0.250)	(0.240)	(0.237)
Mon.Rew. & Pers.Norm x DonationsBehav1	0.071	−0.008	0.010	−0.010
	(0.240)	(0.237)	(0.243)	(0.240)
Mon.Rew. & Soc.Norm x DonationsBehav1	−0.410	−0.502*	−0.263	−0.340
	(0.257)	(0.254)	(0.247)	(0.244)
Personal norm	−13.045	−10.630	−7.655	−2.194
	(21.265)	(20.985)	(19.426)	(19.166)
Social norm	32.669	35.920^{+}	17.670	24.330
	(20.944)	(20.688)	(22.022)	(21.740)
Mon.Rew. & Pers.Norm	−13.097	−8.131	−15.809	−11.088
	(19.627)	(19.377)	(19.941)	(19.682)
Mon.Rew. & Soc.Norm	19.433	22.517	8.369	15.887
	(21.819)	(21.527)	(20.459)	(20.222)
Monetary reward	−21.931	−23.283	−28.021	−23.553
	(20.690)	(20.390)	(19.241)	(19.010)
elicited personal norm	0.277	0.621		
	(1.277)	(1.263)		

Personal norm x elicited personal norm	0.534	0.319		
	(2.045)	(2.019)		
Social norm x elicited personal norm	−3.117	−3.483^{+}		
	(1.932)	(1.909)		
Mon.Rew. & Pers.Norm x elicited personal norm	0.851	0.267		
	(1.880)	(1.857)		
Mon.Rew. & Soc.Norm x elicited personal norm	−2.122	−2.304		
	(2.061)	(2.031)		
Monetary reward x elicited personal norm	0.742	0.939		
	(2.060)	(2.029)		
Personal norm x elicited personal norm x DonationsBehav1	−0.024	−0.016		
	(0.026)	(0.025)		
Social norm x elicited personal norm x DonationsBehav1	0.033	0.040^{+}		
	(0.024)	(0.024)		
Mon.Rew. & Pers.Norm x elicited personal norm x DonationsBehav1	−0.005	0.004		
	(0.023)	(0.023)		
Mon.Rew. & Soc.Norm x elicited personal norm x DonationsBehav1	0.038	0.046^{+}		
	(0.025)	(0.025)		
Monetary reward x elicited personal norm x DonationsBehav1	−0.011	−0.007		
	(0.025)	(0.025)		
elicited personal norm x DonationsBehav1	0.001	−0.005		
	(0.017)	(0.016)		
elicited social norm			−0.209	0.391
			(1.241)	(1.231)
Personal norm x elicited social norm			0.037	−0.476
			(1.791)	(1.767)
Social norm x elicited social norm			−1.761	−2.448
			(2.020)	(1.994)
Mon.Rew. & Pers.Norm x elicited social norm			1.103	0.555
			(1.848)	(1.825)
Mon.Rew. & Soc.Norm x elicited social norm			−1.015	−1.607
			(1.882)	(1.859)
Monetary reward x elicited social norm			1.334	0.948
			(1.825)	(1.805)
Personal norm x elicited social norm x DonationsBehav1			0.0005	0.003
			(0.022)	(0.022)
Social norm x elicited social norm x DonationsBehav1			0.025	0.034
			(0.025)	(0.024)
Mon.Rew. & Pers.Norm x elicited social norm x DonationsBehav1			0.001	0.004
			(0.022)	(0.022)
Mon.Rew. & Soc.Norm x elicited social norm x DonationsBehav1			0.022	0.027
			(0.023)	(0.022)
Monetary reward x elicited social norm x DonationsBehav1			−0.006	−0.001

			(0.022)	(0.022)
elicited social norm x DonationsBehav1			0.003	-0.001
			(0.016)	(0.016)
Constant	101.329***	52.530**	106.205***	55.062***
	(12.937)	(16.205)	(13.199)	(16.513)
Control variables		X		X
Observations	2,196	2,196	2,196	2,196
R^2	0.016	0.062	0.014	0.060
Adjusted R^2	0.006	0.043	0.004	0.042

Note: Table shows donations in behaviour 2 as a dependent variable regressed on treatment effects and interactions with the elicited norms on the extensive margin using an OLS mode. The estimates present the 2SLS-regression. OLS regression in the first stage, regressing *DonationsBehav1* on the donation amount assigned to breaking a code (see bottom part of Table A5 - Column 3 and 4 in Appendix), i.e. *DonationsBehav1* is instrumented by the assigned donation value in behaviour 1 for breaking a code. The treatment indicators, the instrumented variable for amount donated in behaviour 1 and the interaction terms for the treatment indicators and the instrumented variable for amount donated in behaviour 1 are used as independent variables. The sample is restricted to participants donating positive amounts in behaviour 2 (effects on the intensive margins). Robust standard errors in parenthesis, $^{+}p<0.1$; $^{*}p<0.05$; $^{***}p<0.01$

Table 9: Average Codes solved and amounts donated in behaviour 1 and 2 by country

	Dependent variable:	
	CodesSolved	Donations
	(1)	(2)
Poland	−0.999**	27.280+
	(0.330)	(14.541)
Personal Norm	0.217	13.540
	(0.380)	(15.286)
Social Norm	0.153	32.483*
	(0.361)	(15.313)
Monetary Reward	0.087	−4.545
	(0.365)	(14.624)
Mon.Rew. & Pers.Norm	0.354	12.529
	(0.358)	(15.114)
Mon.Rew. & Soc.Norm	0.515	18.032
	(0.376)	(14.701)
Personal Norm x Poland	−0.214	25.144
	(0.522)	(22.558)
Social Norm x Poland	0.055	−17.364
	(0.507)	(22.279)
Monetary Reward x Poland	0.435	−14.539
	(0.499)	(21.424)
Mon.Rew. & Pers.Norm x Poland	−0.196	3.604
	(0.506)	(21.836)
Mon.Rew. & Soc.Norm x Poland	−0.089	−4.343
	(0.513)	(21.321)
Constant	11.852***	11.141
	(0.234)	(10.198)
Observations	3,782	3,782
Log Likelihood	−11,145.630	−9,589.225
Wald Test (df = 11)	47.208***	34.477***
Note:	*p<0.1; **p<0.05; ***p<0.01	

2 Additional analysis

2.1 Identification strategy

To determine how different policy mixes imposed on prosocial behaviour spill over to subsequent prosocial behaviour, we first clarify the taxonomy of spillovers and how to estimate them [1]. For this, we use the following tobit regression model[2].

$$E[y_i|y < 70, y > 0] = \beta_0 + \beta_1 Di,1 + \sum_{j=1} \beta_j X_{i,p} + \sum_{k=1} \beta_k D_{i,1} \cdot X_{i,p} + \beta_c c_i + \sigma\lambda(\alpha)$$

$$y_i \equiv \text{Donations to charity in behaviour 2}$$

$$\hat{D}_{i,1} \equiv \text{Donations in behaviour 1}$$

$$X_{i,p} \equiv \text{Treatment indicators}$$

$$c_i \equiv \text{Control variables}$$

$$\sigma\lambda(\alpha) \equiv \text{Regularisation term}$$

We regress the donations provided in behaviour 2, y_i, on the donations generated by breaking codes within the 6 minutes of behaviour 1, D_i. In the model, the parameter β_1 captures the effect of increases in the donations in behaviour 1 on donations provided in behaviour 2 in the *Prosocial only* treatment. The treatment indicators, $\beta_j X_{i,p}$, measure the effect of the interventions imposed on behaviour 1 on the donations provided in behaviour 2 independently of the level of a prosocial contribution in behaviour 1. Lastly, the interaction terms, $\sum_{k=1} \beta_k \hat{D}_{i,1} \cdot X_{i,k}$, capture the difference in the effect of prosocial contributions in behaviour 1 on donations provided in behaviour 2 in the intervention treatments compared with the *Prosocial only* treatment.

Figure 1 illustrates the regression model and relates its terms to the distinct spillover effects. The x-axis shows the donations in behaviour 1, the y-axis depicts donations in behaviour 2. The two black lines are hypothetical fitted regression lines depicting the relationship between donations in behaviour 1 and behaviour 2 for the *Prosocial only* (upper line) and interventional

[1]The analysis presented here largely follows our preregistered plan, with some refinements. First, we do not employ the first model aimed at assessing the broadly defined spillover effects. This exclusion follows the expanded categorisation of spillover effects, including 'overall spillover effect', its components, and the 'pure spillover effect'. Instead, we employ the second preregistered Tobit regression model and a 2SLS approach to estimate spillover effects, as originally planned.

Second, we introduce a new model - see Section B3- to estimate the newly introduced *overall spillover effect*.

Finally, we do not conduct the sub-sample analysis of treatment effects based on variables such as altruistic preferences, concerns about the environment, trust, gender and age, as initially proposed, but rather use these variables as control factors to account for potential confounding effects.

[2]The regularisation term, denoted as $\sigma\lambda(\alpha)$, is a component added to the regression model to penalise complex or extreme parameter estimates, thus preventing overfitting and improving the model's generalisability (Tibshirani, 1996).

treatments (lower line). The spillover effects comprise the *overall spillover effect*, consisting of the *interventional spillover effect* and the *persistence effect*, and the *pure spillover effect*. The *pure spillover effect*, indicated in the figure by β_1, captures how donations in behaviour 1 affect the willingness to donate in behaviour 2 without any intervention. The *overall spillover effect* is determined by the yellow area in Figure 1 and represents the broadest measurement of spillover effects [3]. The *interventional spillover effect*, β_k, captures the intervention-induced change in the degree to which prosocial actions in behaviour 1 affect the engagement in behaviour 2. It is determined by the difference in slopes of the intervention treatments α_k and the *pure spillover effect*, β_1. Lastly, the *persistence effect*, β_j, captures the effect of an intervention targeting behaviour 1 on the willingness of individuals to engage in behaviour 2 independently of their contributions in behaviour 1 (no prosocial contribution opportunity in behaviour 1). It is measured by the difference in the intercepts of the regression lines of the intervention treatments compared with the no-intervention treatment [4].

[3]To measure the *overall spillover effect*, we rely on a tobit model, which does not include interaction terms between donations in behaviour 1 and donations in behaviour 2 (see section B3).

[4]Although the different spillover effects are related to each other, there is no direct mathematical relation from which to compute the *overall spillover effect* from the *pure spillover effect*, the *interventional spillover effect*, and the *persistence effect*. The *overall spillover effect* depends on the distribution of donations in behaviours 1 and 2 in the prosocial and interventional treatments and is estimated in a separate model (see Section B3). The *pure spillover effect*, the *interventional spillover effect*, and the *persistence effect* instead are obtained from fitted regression lines originating from the model presented in this section.

Figure 1: Taxonomy of spillover effects

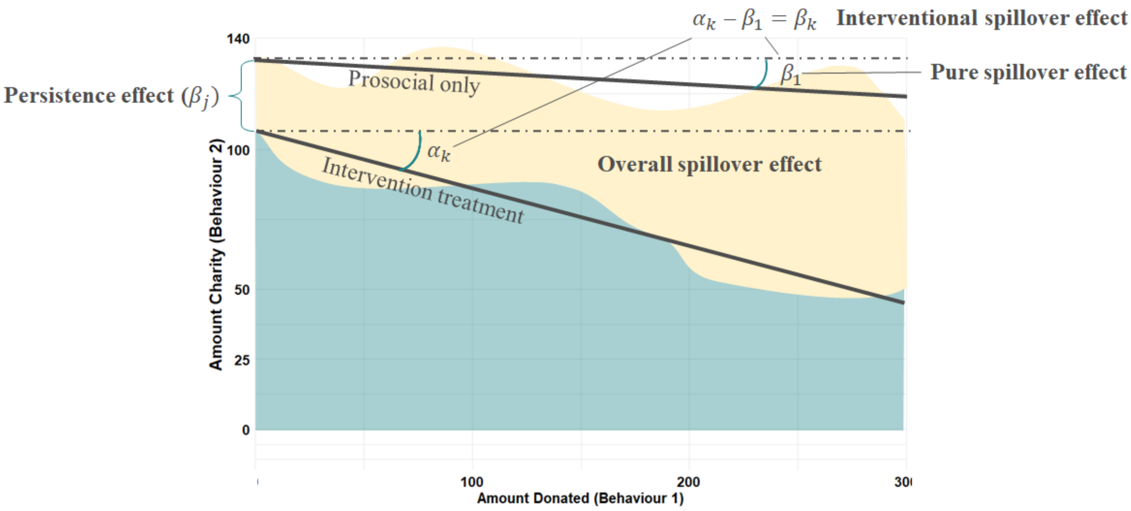

Note: The graph serves as an example illustration of the *pure spillover effect* and the *overall spillover effect* (consisting of the *interventional spillover effect* and the *persistence effect*). It displays the stylised fitted regression lines of the *prosocial only* and intervention treatments, and shows the correlation between donations in behaviour 1 (x-axis) and behaviour 2 (y-axis). There is no direct mathematical relation between the *overall spillover effect* and the other effects, since the yellow area is not necessarily enclosed by the regression lines, and is likely to follow a nonlinear course.

The identification strategy for the *pure* and *interventional spillover effects* relies on the correlation between behaviour 1 and behaviour 2. However, this correlation is likely to be driven by endogenous factors. In particular, some omitted variables, such as personal characteristics and preferences, might determine donation decisions in behaviour 1 and behaviour 2. Therefore, we rely on an approach that introduces exogenous variation in contributions in behaviour 1 and use an instrumental variable approach to estimate the *pure spillover effect* and the *interventional spillover effects*. We implement exogenous variation in the contributions to charity in behaviour 1 by randomly assigning different values donated by participants once they break a code. These values range from 0 to 12 survey points in step two. We use this variable in a 2SLS regression design, in which we regress the total donations generated by breaking codes within the 6 minutes of behaviour 1, D_i, on the specific donation value assigned to participants for breaking a code in behaviour 1, $Z_{i,1}$ in the first stage of the regression, $\hat{D}_i = \beta_0 + \beta_1 Z_{i,1} + \varepsilon_i$. We use the fitted

values from this regression, $\hat{D}_i$, as an instrument in the second stage, as follows:

$$E[y_i|y<70, y>0] = \beta_0 + \beta_1 \hat{D}_{i,1} + \sum_{j=1} \beta_j X_{i,p} + \sum_{k=1} \beta_k \hat{D}_{i,1} \cdot X_{i,p} + \beta_c c_i + \sigma\lambda(\alpha)$$

$$\begin{aligned} y_i &\equiv \text{Donations to charity in behaviour 2} \\ \hat{D}_{i,1} &\equiv \text{Estimated donations in behaviour 1} \\ X_{i,p} &\equiv \text{Treatment indicators} \\ c_i &\equiv \text{Control variables} \\ \sigma\lambda(\alpha) &\equiv \text{Regularisation term} \end{aligned}$$

We argue that the instrument fulfills the exclusion restriction, as the exogenously assigned donation in behaviour 1 can affect the donation decision in behaviour 2 only by means of the spillover effect. Thus, in the second stage of the regression, we estimate the correlation between the effect of the exogenously imposed variation in the donations in behaviour 1, $\hat{D}_i$ and the corresponding effect on behaviour 2, y_i. Thus, this estimate is independent of endogenous factors.

2.2 Utility framework

We extend the utility framework from Picard (2023), aiming to describe the key psychological mechanisms underlying spillovers. The framework comprises two periods, $t = [1,2]$, and agents, $i = [1,...,n]$ who can engage in a prosocial activity, $a_{i,t}$, at the cost of C_t, using endowment I. The framework includes the potential effects of interventions that target (1) personal norms, represented by the agent's perception of appropriate behaviour, $g_{i,t}$, (2) social norms, reflecting collective perceptions of appropriate behaviour (i.e. beliefs that others believe that a certain behaviour is appropriate), $s_{i,t}$, and (3) monetary considerations, $b_{i,t}$. Since we aim to investigate spillover effects of interventions, we consider interventions to have taken place only in the period preceding the behaviour, $t-1$. The agent's decision-making process is described by the following utility function:

$$U_i(a_{i,t-1}, a_{i,t}) = I + \sum_{t=1}^{2} [w_t(a_{i,t}, g_{i,t}) + m_t(a_{i,t}, s_{i,t}) + v_t(a_{i,t}, A_{i,t})] - \frac{C_{t-1}(a_{i,t-1})}{b_{t-1}} - C_t(a_{i,t}) \quad (1)$$

where:

- $w_t(a_{i,t}, g_{i,t})$ represents the utility derived from personal norm compliance. It thus denotes personal norm compliance as a motive to engage in prosocial activities. This compliance is influenced by (1) the disutility obtained from the gap between the agent's behaviour,

$a_{i,t}$, and personal perceptions of appropriateness, $g_{i,t}$, (2) having previously received a sanction or a reward $b_{i,t-1}$.

- $m_t(a_{i,t}, s_{i,t})$ captures the utility from social norm compliance. It thus denotes adherence to social norms as a motive to engage in prosocial activities. This compliance is determined by (1) the disutility obtained from the gap between the agent's behaviour, $a_{i,t}$, and collective perceptions of appropriateness, $s_{i,t}$, and (2) past beliefs that others believe that a certain behaviour is appropriate, $s_{i,t-1}$, and (3) having previously received a sanction or a reward $b_{i,t-1}$.
- $v_t(a_{i,t}, A_{i,t})$ denotes the utility from accumulated prosocial actions, where $A_{i,t}$ represents the history of prosocial behaviours influencing current decisions.

When faced with opportunities for prosocial behaviour, individuals assess whether their accumulated history of good conduct reflects compliance with personal norms, social norms, or incentives.

2.3 Estimation model of the overall spillover effect

In the model, the treatment indicators, $X_{i,k}$, which enable us to evaluate $y_k - y_1$ (the difference in donations in the intervention treatments and the donation level in the Prosocial only treatment), provide us with the *overall spillover effect*.

$$E[y_i | y < 70, y > 0] = \beta_0 + \beta_1 x_{i,1} + \sum_{k=1} \beta_j X_{i,j} + \sum_{k=1} \beta_k x_{i,1} \cdot X_{i,k} + \beta_c c_i + e_i$$

$$y_i \equiv \text{Donations to charity in behaviour 2}$$

$$x_{i,1} \equiv \text{Donations in behaviour 1}$$

$$X_{i,k} \equiv \text{Treatment indicators}$$

$$c_i \equiv \text{Control variables}$$

3 Script of Experiment

Background information

Country

1. Italy Italian
2. Poland Polish

Treatment (experiment conditions or treatment interventions).

2. Pro-social only
3. Personal Norm Nudge
4. Social Norm Nudge
5. Monetary Reward
9. Monetary Inc. & Pers. Norm Nudge
10. Monetary Inc. & Soc. Norm Nudge

Treatment		N=3745 (for IT and PL together)	IT (n=1875)	PL (n=1870)
2	Pro-social only	800	400	400
3	Personal Norm Nudge	589	295	294
4	Social Norm Nudge	589	295	294
5	Monetary Reward	589	295	294
9	Monetary Inc. & Pers. Norm Nudge	589	295	294
10	Monetary Inc. & Soc. Norm Nudge	589	295	294

Scripter recode Treatment into **Intervention_type**

1. No intervention (if Treatment=1-2)
2. Single intervention (if Treatment=3-8)
3. Policy mixes (if Treatment=9-12)

Scripter recode Treatment into **Intervention_type2**

1. Control (if Treatment=1)
2. Pro-social only (if Treatment=2)
3. Monetary Incentive (if Treatment=5, 6)
4. Nudge-treatments (if Treatment=3, 4, 7, 8)
5. Monetary Incentive + Nudge (if Treatment=9, 10, 11,12)

**Amount2**	**Within each treatment x country**
If treatment=2 (pro-social only)	
0 points	34% of sample
2 points	11% of sample
4 points	11% of sample
6 points	11% of sample

8 points	11% of sample
10 points	11% of sample
12 points	11% of sample
If treatment<>2 (not pro-social only)	
0 points	16% of sample
2 points	14% of sample
4 points	14% of sample
6 points	14% of sample
8 points	14% of sample
10 points	14% of sample
12 points	14% of sample

Introduction text

Base: all respondents

Intro

Welcome!

Thank you for your interest in this study, which is being conducted as part of a research project across the European Union. The study has been designed and funded by the European Commission and is being conducted by Ipsos. It will take about 30 minutes to complete.

Please read all the information and answer the questions carefully.

In addition to other topics, we will ask you a few questions related to your political opinion. Rest assured that a "Prefer not to say" option will always be available if you feel uncomfortable answering such questions. Your answers throughout this survey will be kept confidential. The European Commission will not receive any information that would allow you to be identified. Your responses will be grouped together with the responses provided by all participants and your personal data will be held for no longer than 12 months. Your answers will be used strictly for research purposes and may be used to inform policy. Anonymised data from this survey – without any of your personal information included – may be made publicly available for further research.

Participation in the survey is voluntary and you may withdraw consent at any time.

Do you agree to participate given the above conditions?

1. **Yes – I have read the information above and agree to take part in the survey.**
2. **No – I do not agree to take part in this survey.** **[screen out]**

Screener

Base: all respondents

GENDER_NONBINARY_. Are you…?

- _1 Male
- _2 Female
- _3 Other
- _4 Prefer not to answer

Scripter: IF GENDER_NONBINARY = 4: SCREEN OUT

Scripter: recode answer into D1

Scripter: if Gender_Nonbinary = 3, allocate to least filled category 1 or 2 for quota setting

Base: all respondents

[Standard Screener: DO NOT MODIFY OR TRANSLATE]

YEAR/MONTH. What is your date of birth?

- YEAR
- _1910 1910
- ...
- _2015 2015
- MONTH
- _1 January
- _2 February
- _3 March
- _4 April
- _5 May
- _6 June
- _7 July
- _8 August
- _9 September
- _10 October
- _11 November
- _12 December

[Standard Screener: DO NOT MODIFY OR TRANSLATE]

QUOTAGERANGE [Hidden]. Hidden Question - QUOTAGERANGE "this is a dummy question that will hold age breaks" for the quotas that should be defined by the PM; it CAN be edited and lines can be added to meet survey objectives.

- _18_34 "18-34",

- _35_54 "35-54",
- _55_ "55+"

[TERMINATE IF LESS THAN 18]

[Standard Screener: DO NOT MODIFY OR TRANSLATE]

RESP_AGE [Hidden]. Hidden Question - RESP_AGE "this is a dummy question that will hold age"

- USE RESP_AGE [Hidden] response list

[Standard Screener: DO NOT MODIFY OR TRANSLATE]

Scripter: recode age into D2

Scripter: recode D2 into D2_cat:

1. 18-24
2. 25-34
3. 35-44
4. 45-54
5. 55-64
6. 65+

Base: all respondents

D3 [S]

In which region do you live?

Scripter: insert country specific lists: See Excel D3 Region

Scripter: include RecodeRegion based on D3 (see Excel D3 Region)

98. Don't know
99. Prefer not to answer

Scripter: IF D3 = 98 OR 99: SCREEN OUT

Base: all respondents

D4 [S]

What is the highest level of school you have completed, or the highest degree you have received?

Scripter: insert country specific lists: See Excel D4 Education

Scripter: include ISCED based on D5 (see Excel D4 Education)

98. Don't know
99. Prefer not to answer

Main Questionnaire

Part I

Base: all respondents

Info1 [infoscreen]

This study is about individual attitudes and behaviors under various circumstances. It is structured in **two parts**.

- **Part I** will take about **12 minutes**
- **Part II** will take about **10 minutes**

Throughout the study, you will be able to **gain additional survey points** by various tasks on top of your participation fee. Your additional earnings (on top of your participation fee) will be calculated in points. At the end of the survey, the total amount of points you have earned will be converted into real money at the following rate: <if country=1: 100 points = 1€, if country=2: 100 points = 2.6 złoty>.

Scripter: for Amount2=0, show: In part I, you will have the opportunity to work on a specific task.
Scripter: for Amount2>0, show: In part I, you have the opportunity to contribute to charity through participating in a task.

In part II, we ask you to answer various questions.

Base: all respondents

Info2 [infoscreen]

In the following, you **proceed with part I**.

This part of the survey will be structured as follows:

1. **Explanation** of the task.
2. A **test round** of the task
3. **6 minutes** of conducting the **task**

Base: all respondents

Info3 [infoscreen]

This task consists of two modes:

- The automatic mode
- The work mode

Please note: initially, the task starts in the work mode. You can switch back and forth between work and automatic mode as often as you like by clicking on a button.

The automatic mode

Scripter: if Amount2 = 0, show:

The automatic mode is a **passive mode**, in which you do not need to be active. You can simply wait until the 6 minutes will have passed. Thus, you can remain in that mode **as long as you desire**. While being in this mode, the **time will continue to run**. During that time you cannot work on the task.

The picture below provides an example of the automatic mode’s layout.

Scripter: insert image info3a.

Scripter: if Amount2 > 0, show:

The automatic mode is a **passive mode**, in which you do not need to be active. You can simply wait until the 6 minutes will have passed. Thus, you can remain in that mode **as long as you desire**. While being in this mode, the **time will continue to run**. During that time you cannot work on the task. For **every 10 seconds** of a break, we will **transfer <Amount3> to <if country=1: Banco Alimentare, if country=2: Banki Żywności>**.

The picture below provides an example of the automatic mode’s layout.

Scripter: insert image info3b.

<if country=1: insert image info6_IT>

<if country=2: insert image info6_PL>

<if country=1: Banco Alimentare, if country=2: Banki Żywności> **prevents food waste** by distributing qualitatively flawless food that cannot be used anymore in the economic process **to people in need**. <if country=1: Banco Alimentare, if country=2: Banki Żywności> works against throwing away food, therefore **saving environmental resources** and **fighting against poverty** at the same time.

Scripter, for Amount2 >0: if participants click on the "Take a Break" button, the amount donated shall increase by amount3 every 10 seconds.]

Amount3 is a fraction of amount2 (amount2/20) and should be assigned for every 10 seconds they take a break (*either 0points, 0.1points, 0.2points, 0.3points, 0.4points, 0.5points or 0.6points)* the same way as amount2 is assigned.

Scripter:

**Amount3**	**same assignment as amount2**
0 points	If Amount2=0 point
0.1 points	If Amount2=2 point
0.2 points	If Amount2=4 points
0.3 points	If Amount2=6 points
0.4 points	If Amount2=8 points
0.5 points	If Amount2=10 points
0.6 points	If Amount2=12 points

The work mode

In the work mode, you have the option to **decode codes** in a time range of **6 minutes**. To solve one randomly chosen **seven-digit numerical code** you need to **match the digits with the seven corresponding letters** provided in the translation table.

Scripter: if Amount2 = 0, show:

Solving a code correctly has **no material consequences** neither for yourself nor for others.

Scripter: if Amount2 > 1 (not control group), show:

For each code that you solve correctly within the 6 minutes of the task, we will make a **monetary transfer to <if country=1: Banco Alimentare, if country=2: Banki Żywności>**.

The amount of this transfer has been randomly determined. In your case, **each solved code leads to a donation of <amount2>**.

Scripter: **amount2** (*either 0, 2, 4, 6, 8, 10 or 12 points)* should be assigned randomly within each treatment and within each country so we have an equal distribution.

**Amount2**	**Within each treatment x country**
If treatment=2 (pro-social only)	
0 points	34% of sample
2 points	11% of sample
4 points	11% of sample
6 points	11% of sample
8 points	11% of sample
10 points	11% of sample
12 points	11% of sample
If treatment<>2 (not pro-social only)	
0 points	16% of sample
2 points	14% of sample
4 points	14% of sample
6 points	14% of sample
8 points	14% of sample
10 points	14% of sample
12 points	14% of sample

---

*Please note that we provide a proof of this transfer on request. The proof does **not** suffice to be included in the tax declaration.

In the **below image** you can see an example of the task.

In the center of the screen, there is a **numerical code**.

In the upper part of the screen there is a **translation table**, which indicates the letters that correspond to the respective numbers within the numerical code. This table will be shown to you throughout the task and its **content will renew with each code**.

In the lower part, there is the **answer field** where you enter your translation. You can confirm your input with the "Next" button .

Scripter: Example image (to be updated with final script – in local language)

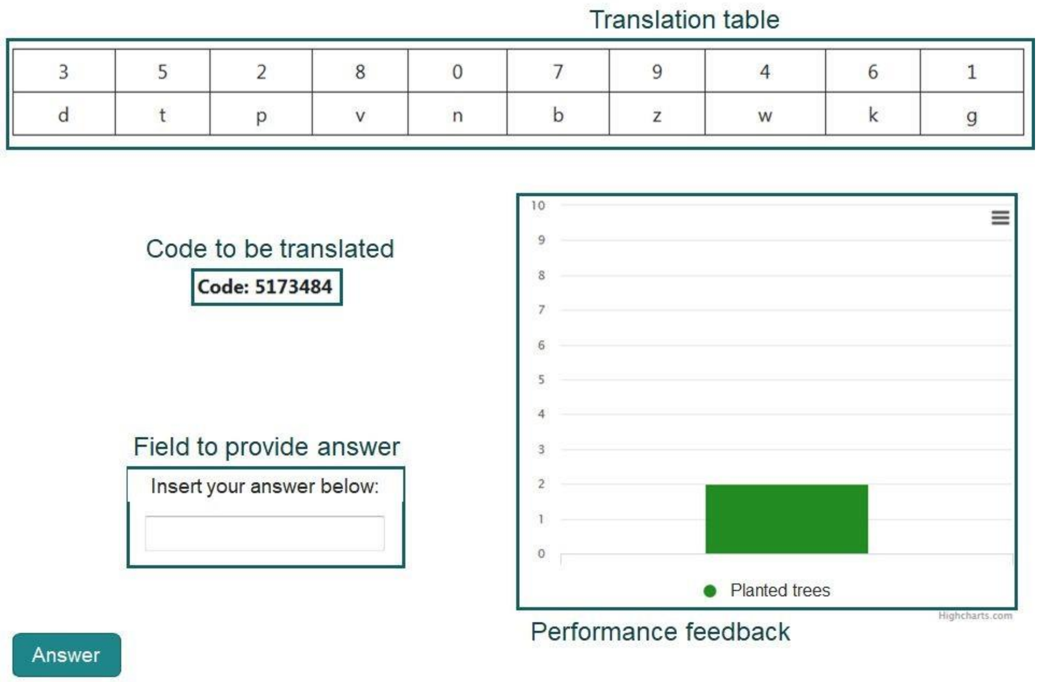

Base: all respondents

Info4 [infoscreen]

In the **below picture** the solution steps are shown.

The **numbers in the numerical code** must be assigned to their **respective letter in the translation table**. Attention: the numbers and letters are in a random order.

This letter must then be entered in the input field below.

Scripter: Example image (to be updated with final script – in local language)

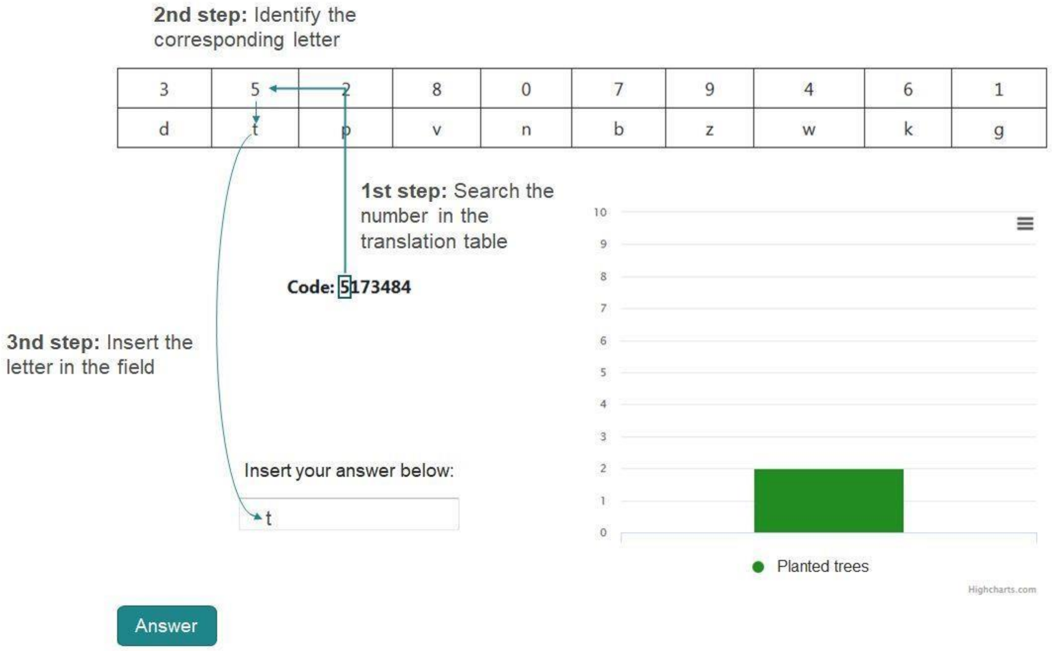

Base: all respondents

Info5 [infoscreen]

In the **below image**, all numbers in the numerical code have been translated and correctly entered into the solution field.

In case the **answer was not entered correctly**, an **error message** will be displayed and you will be asked to correct the answer. You have **three attempts** to enter the correct translation of the code.

If the answer is correct or if you have entered three wrong answers in a row, a new code will appear to be solved.

Scripter: Example image (to be updated with final script – in local language)

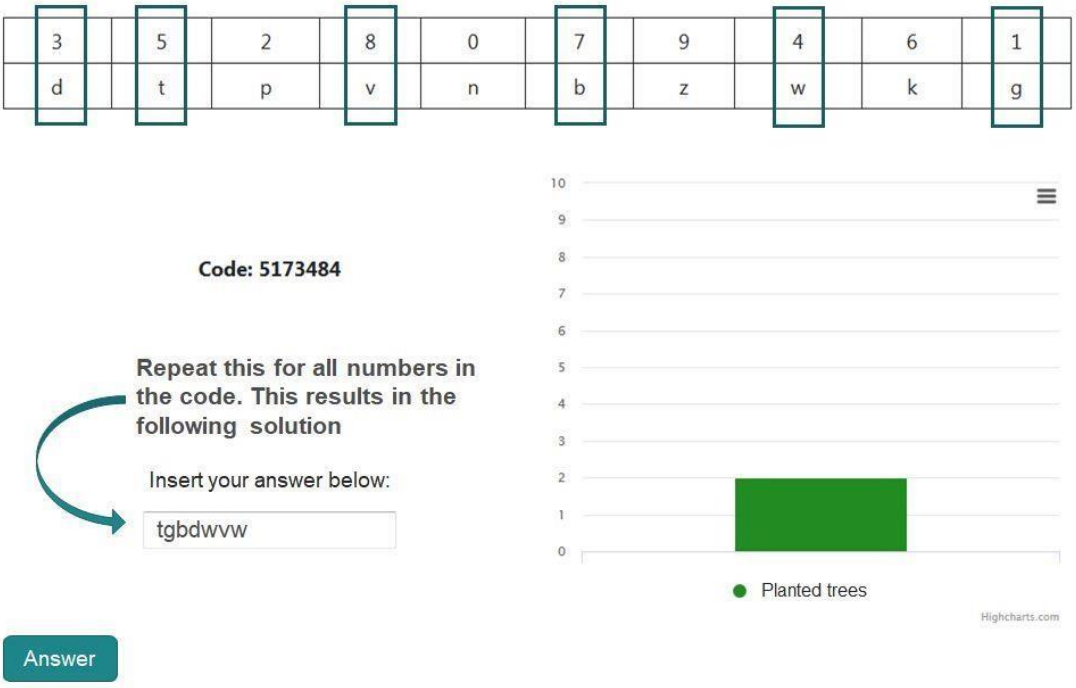

Base: all respondents

Coding1 [infoscreen]

To make sure everything is clear, you will now be shown a test round of the decoding task. We kindly ask you to translate numerical codes into letters, by using the translation table at the top of the screen. You can also test how to switch to the automatic mode and back by means of the button.

After that, you will receive some questions to make sure everything is clear and then the 6 minutes of the task will start. The remaining time will be always visible to you.

Base: all respondents

CodingTest1 [O – 7 digits]

Here is the first test code.

You have the option to either be in the automatic mode or in the work mode in which you can translate a sequence of numbers into letters. You can see below:

- a sequence of numbers,
- a translation-table that specifies by which letters the numbers are represented, and
- a text field in which the solution is to be entered.

Please enter the solution in the input field.

Scripter: insert coding task, code 1, similar layout as image on Info3 – as a test, so no timer yet. Show translation table image Coding_table41.jpg

If answer is correct show screen “Your answer is correct!” and go to next code, if answer is wrong show error message and they can try again: “Your answer is incorrect. Please try again.” For each code, a respondent can give 3x a wrong answer, then move to the next code. For this 3rd attempt, show error message: “Your answer is incorrect. The correct answer is: <insert correct answer of that code>. You can now proceed to the next code”.
Scripter: add variable that captures how often the question was answered to be able to analyse nr of wrong attempts.

Code nr	Code	Correct answer
41	3520167	fxdirmg

Base: If treatment = 3 [Treatment = PersNorm]

Info7 [infoscreen]

An **average person** in the task can decide themselves how **<if Amount2=0>** much effort to provide to solve the codes (see Table below). **<if Amount2>0>** much s/he wants to contribute to <if country=1: Banco Alimentare, if country=2: Banki Żywności> by **providing more or less effort to solve the codes** (see Table below).

If the person puts **maximum effort**, s/he is capable of solving 13 codes **<if Amount2>0>**and **donates <XY_high>** respectively.

If the person engages in solving codes, but provides **medium effort**, she/he solves 10 codes **<if Amount2>0>** and **donates <XY_medium>**.

If the person provides **minimum effort**, she/he will take a break the entire 6 minutes **<if Amount2>0>** and **contribute <XY_low>**.

	**Codes solved**	**Donation Scripter: Skip column if Amount2=0**
Maximum effort	13	<XY_high>
Medium effort	10	<XY_medium>
Minimal effort	0	<XY_low>

Scripter: also insert the above table on the screen. The amount to insert in XY_low, XY_medium, XY_high depends on the amount assigned in Amount2:

XY_low	LFQ priorities
4 points	If Amount2=2 points
7 points	If Amount2=4 points
11 points	If Amount2=6 point
14 points	If Amount2=8 points
18 points	If Amount2=10 points
22 points	If Amount2=12 points

XY_medium	LFQ priorities
20 points	If Amount2=2 points
40 points	If Amount2=4 points
60 points	If Amount2=6 point
80 points	If Amount2=8 points
100 points	If Amount2=10 points
120 points	If Amount2=12 points

XY_high	LFQ priorities
26 points	If Amount2=2 points
52 points	If Amount2=4 points
78 points	If Amount2=6 point
104 points	If Amount2=8 points
130 points	If Amount2=10 points
156 points	If Amount2=12 points

Base: If treatment = 3 [Treatment = PersNorm]

Q1 [S - slider]

In the following, we would like you to evaluate, **according to your own opinion** and independently of the opinion of others, which of the following numbers of codes would be **appropriate** to solve in the task. “Appropriate” behavior means the behavior that you **personally consider to be “correct” or “moral”**. The standard is, hence, your personal opinion, independently of the opinion of others.

We kindly ask you to answer as precisely as possible with your own honest opinion. There is **no right or wrong answer**; you will not get any additional survey points depending on your answer.

I think that the appropriate number of solved codes for this task would be:

Scripter insert slider with left anchor: 0 codes and right anchor 25, also show the selected amount

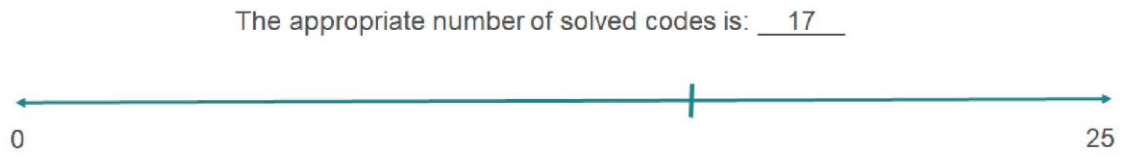

Low effort | Medium effort | High effort

Base: If treatment = 4 [Treatment = SocNorm]

Info8 [infoscreen]

An **average person** in the task can decide themselves how **<if Amount2=0>** much effort to provide to solve the codes (see Table below). **<if Amount2>0>** much s/he wants to contribute to <if country=1: Banco Alimentare, if country=2: Banki Żywności> by **providing more or less effort to solve the codes** (see Table below).

If the person puts **maximum effort**, she/he is capable of solving 13 codes **<if Amount2>0>**and **donates <XY_high>** respectively.

If the person engages in solving codes, but provides **medium effort**, she/he solves 10 codes **<if Amount2>0>** and **donates <XY_medium>**.

If the person provides **minimum effort**, she/he will take a break the entire 6 minutes **<if Amount2>0>** and **contribute <XY_low>**.

	**Codes solved**	**Donation Scripter: Skip column if Amount2=0**
Maximum effort	13	<XY_high>
Medium effort	10	<XY_medium>
Minimal effort	0	<XY_low>

Scripter: also insert the above table on the screen. The amount to insert in XY_low, XY_medium, XY_high depends on the amount assigned in Amount2 (see table of info7).

Base: If treatment = 4 [Treatment = SocNorm]

Q2 [S - slider]

In the following, we would like you to guess which of the following numbers of codes **other participants rated as the appropriate** number to solve in the task, independent of your own opinion on the appropriate behavior. "Appropriate" behavior means the behavior that **others consider to be "correct" or "moral".** The standard is, hence, the opinion of others, independently of your personal opinion.

We kindly ask you to answer as precisely as possible. If your **guess is correct, you will gain 10 additional survey points. If your guess is incorrect, you will not gain any additional points for your guess.**

I think that other participants rated on average the appropriate number of solved codes for this task to be: Scripter: insert slider with left anchor: XY_Low and right anchor XY_high.

Give points to respondents depending on correct value (see table below) and their answer.
If respondent's answer=Correct value, award 10 points.

**Correct value**
11 codes

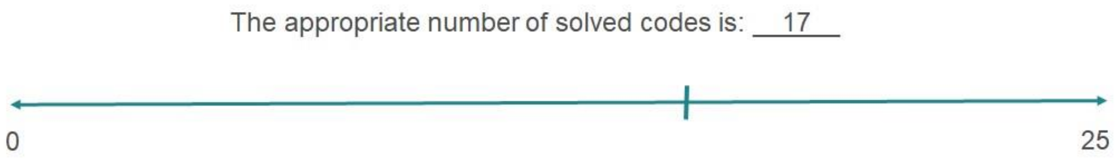

Low effort Medium effort High effort

Base: If treatment = 5 [Treatment = MonReward]

Info9 [infoscreen]

Scripter: Participants receive a reward of 10 points for correctly answering codes.

Please note that the **number of codes solved in the work mode will affect your additional survey points**. In addition to the donation generated **for each correctly answered code**, you will **gain 10 points**, which will be added to your participation fee. For example, if you solve 5 tasks correctly, you will earn an additional amount of 50 points. If you solve 10 tasks correctly, you will earn an additional amount of 100 points.

Base: If treatment = 9 [Treatment = PersNorm x MonReward]

Info13 [infoscreen]

An **average person** in the task can decide themselves how **<if Amount2=0>** much effort to provide to solve the codes (see Table below). **<if Amount2>0>** much s/he wants to contribute to <if country=1: Banco Alimentare, if country=2: Banki Żywności> by **providing more or less effort to solve the codes** (see Table below).

If the person puts **maximum effort**, she/he is capable of solving 13 codes **<if Amount2>0>**and **donates <XY_high>** respectively.

If the person engages in solving codes, but provides **medium effort**, she/he solves 10 codes **<if Amount2>0>** and **donates <XY_medium>**.

If the person provides **minimum effort**, she/he will take a break the entire 6 minutes **<if Amount2>0>** and **contribute <XY_low>**.

	**Codes solved**	**Donation Scripter: Skip column if Amount2=0**
Maximum effort	13	<XY_high>
Medium effort	10	<XY_medium>
Minimal effort	0	<XY_low>

Scripter: also insert the above table on the screen. The amount to insert in XY_low, XY_medium, XY_high depends on the amount assigned in Amount2 (see table of info7).

Base: If treatment = 9 [Treatment = PersNorm x MonReward]

Q3 [S - slider]

In the following, we would like you to evaluate, **according to your own opinion** and independently of the opinion of others, which of the following numbers of codes would be **appropriate** to solve in the task. “Appropriate” behavior means the behavior that you **personally consider to be “correct” or “moral”**. The standard is, hence, your personal opinion, independently of the opinion of others.

We kindly ask you to answer as precisely as possible with your own honest opinion. There is **no right or wrong answer**; you will not get any additional survey points depending on your answer.

I think that the appropriate number of solved codes for this task would be:

Scripter insert slider with left anchor: 0 codes and right anchor 25, also show the selected amount

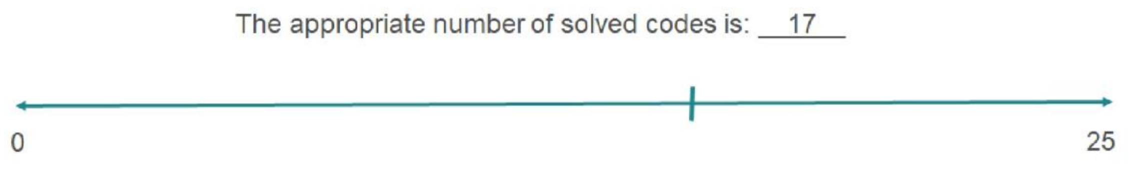

Low effort Medium effort High effort

Base: If treatment = 9 [Treatment = PersNorm x MonReward]

Info14 [infoscreen]

Scripter: Participants receive a reward of 10 points for correctly answering codes.

Please note that the **number of codes solved in the work mode will affect your additional survey points**. In addition to the donation generated **for each correctly answered code**, you will **gain 10 points**, which will be added to your participation fee. For example, if you solve 5 tasks correctly, you will earn an additional amount of 50 points. If you solve 10 tasks correctly, you will earn an additional amount of 100 points.

Base: If treatment =10 [Treatment = SocNorm x MonReward]

Info15 [infoscreen]

An **average person** in the task can decide themselves how **<if Amount2=0>** much effort to provide to solve the codes (see Table below). **<if Amount2>0>** much s/he wants to contribute to <if country=1: Banco Alimentare, if country=2: Banki Żywności> by **providing more or less effort** (see Table below).

If the person puts **maximum effort**, she/he is capable of solving 13 codes **<if Amount2>0>**and **donates <XY_high>** respectively.

If the person engages in solving codes, but provides **medium effort**, she/he solves 10 codes **<if Amount2>0>** and **donates <XY_medium>**.

If the person provides **minimum effort**, she/he will take a break the entire 6 minutes **<if Amount2>0>** and **contribute <XY_low>**.

	**Codes solved**	**Donation Scripter: Skip column if Amount2=0**
Maximum effort	13	<XY_high>
Medium effort	10	<XY_medium>
Minimal effort	0	<XY_low>

Scripter: also insert the above table on the screen. The amount to insert in XY_low, XY_medium, XY_high depends on the amount assigned in Amount2 (see table of info7).

Base: If treatment = 10 [Treatment = SocNorm x MonReward]

Q4 [S - slider]

In the following, we would like you to guess which of the following numbers of codes **other participants rated as the appropriate** number to solve in the task, independent of your own opinion on the appropriate behavior. “Appropriate” behavior means the behavior that **others consider to be “correct” or “moral”**. The standard is, hence, the opinion of others, independently of your personal opinion.

We kindly ask you to answer as precisely as possible. If your **guess is correct, you will gain 10 additional survey points. If your guess is incorrect, you will not gain any additional points for your guess.**

I think that other participants rated on average the appropriate number of solved codes for this task to be: Scripter: insert slider with left anchor: XY_Low and right anchor XY_high.

Give points to respondents depending on correct value (see table below) and their answer.
If respondent's answer=Correct value, award 10 points.

**Correct value**
11 codes

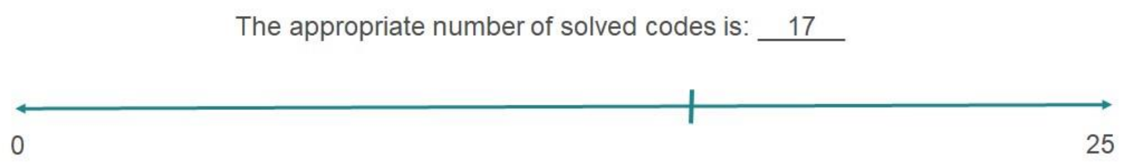

Low effort Medium effort High effort

Base: If treatment = 10 [Treatment = SocNorm x MonReward]

Info16 [infoscreen]

Scripter: Participants receive a reward of 10 points for correctly answering codes.

Please note that the **number of codes solved in the work mode will affect your additional survey points**. In addition to the donation generated **for each correctly answered code**, you will **gain 10 points**, which will be added to your participation fee. For example, if you solve 5 tasks correctly, you will earn an additional amount of 50 points. If you solve 10 tasks correctly, you will earn an additional amount of 100 points.

Base: all respondents

Q5 [Q] (range 0-999)

Before the task starts, we would like to kindly ask you to **guess how many codes do you think you will be able to solve within the 6 minutes** of the task? Please indicate the expected number below.

_____________ codes

Base: all respondents

Info19 [infoscreen]

Before the actual task starts, we would like to ensure that the instructions are clear to you.

Base: all respondents

Q6 [S]

How much time do you have to solve the task?

1. 5 minutes
2. 6 minutes
3. 8 minutes

Scripter: correct answer is 2. If wrong answer given, show error message, and continue: Your answer is incorrect. The correct answer is 6 minutes.

Base: all respondents

Q7 [S]

Scripter: if Amount2=0, show:

What are the consequences of solving one task correctly?

1. No consequences
2. Donation to charity
3. Increase of my additional survey points

Scripter: correct answer depends on Treatment. If wrong answer given, show error message: If Amount2=0 and Q7>1: Your answer is incorrect. The correct answer is "No consequences"

Scripter: if Amount2>0show:

What are the consequences of solving one task correctly?

1. No consequences
2. Donation to <if country=1: Banco Alimentare, if country=2: Banki Żywności>
3. Increase of my additional survey points
4. Donation to <if country=1: Banco Alimentare, if country=2: Banki Żywności> and an increase of my additional survey points

Scripter: correct answer depends on Treatment. If wrong answer given, show error message:
If Treatment=2,3,4,7,8 and Q7<>2 Your answer is incorrect. The correct answer is "Donation to charity"
If Treatment=5,9,10,11 and Q7<4 : Your answer is incorrect. The correct answer is "Donation to charity and Increase of my additional survey points"

Base: all respondents

Q8 [S]

Scripter: if Amount2=0:

What happens when being in the automation mode during the task?

1.

- I cannot work on the task
- the time will be halted

2.
- I can still work on the task

3.
- I cannot work on the task
- the time continues to run

Scripter: correct answer is 3. If wrong answer given, show error message. Your answer is incorrect. The correct answer is “I cannot work on the task and the time continues to run”

Scripter: if Amount2>0:
What happens when being in the automation mode during the task?

1.
- I cannot work on the task
- for each ten seconds of a break, a donation of <Amount3> to <if country=1: Banco Alimentare, if country=2: Banki Żywności> is generated
- the time will be halted

2.
- I cannot work on the task
- the time will be halted

3.
- I cannot work on the task
- for each ten seconds of a break, a donation of <Amount3> to <if country=1: Banco Alimentare, if country=2: Banki Żywności> is generated
- the time continues to run

Scripter: correct answer is 3. If wrong answer given, show error message:
Your answer is incorrect. The correct answer is “I cannot work on the task, for each ten seconds of a break, a donation of <Amount3> to <if country=1: Banco Alimentare, if country=2: Banki Żywności> is generated and the time continues to run”

Base: all respondents

Info20 [infoscreen]
The 6 minute of the task will now start.

Base: all respondents

Coding_task1 till Coding_task40 [O – 7 digits]
You have the option to either be in the automatic mode or in the work mode in which you can translate a sequence of numbers into letters. You can see below:
- a sequence of numbers,
- a translation-table that specifies by which letters the numbers are represented, and
- a text field in which the solution is to be entered.

If the task has been answered correctly or after three incorrect attempts, a new task will appear.
Scripter: insert coding task, start from Code 1 till 40, similar layout as image on Info3.

Show translation table image Coding_table1.jpg till Coding_table40.jpg

If answer is correct go to next code, if answer is wrong show error message and they can try again: "Your answer is incorrect. Please try again."
For each code, a respondent can give 3x a wrong answer, then move to the next code. For this 3rd attempt, show error message: "Your answer is incorrect. The correct answer is: <insert correct answer of that code>. You can now proceed to the next code".
Scripter: for each code add variable that captures how often the question was answered to be able to analyse nr of wrong attempts.

Repeat for max 40 codes (Coding_task1 until Coding_task40) – respondents are to continue until the 6 minutes timer runs out.

Code nr	Code	Correct answer
1	3619872	pxjtaow
2	2514876	vkxmbrg
3	7324895	yudexva
4	4751260	itmrazo
5	9320547	ypkxdlm
6	0137956	nusdcqk
7	5872091	dcgwriq
8	7846310	oiavftx
9	1062347	hmniblq
10	8072534	hmzltps
11	1708352	enlpakx
12	7682940	quxnivr
13	6312578	sahyxjb
14	5107689	bivufoa
15	5720684	yvxfrmt
16	9271364	vlcfkxn
17	2034586	grqmhaf
18	2405819	ntorsqj
19	1256430	xztqpik
20	3760598	ikxfzyr
21	2351796	vrofkpn
22	4672098	olsctna
23	2798416	zcisgae
24	7589163	ypxuwql
25	6729540	xetwhcz
26	3418752	nxbpqrv
27	3827615	vhmxqae
28	8109573	unpbxjc
29	2547396	nrkfidw

30	3785916	iwhgnyx
31	0724895	xbtnymq
32	1608574	odrntum
33	6982035	dsatigp
34	4270358	vbzqfwo
35	2176934	mjwqrzx
36	2936501	kdnruzo
37	7510986	tbqdxhj
38	4507629	qnieglp
39	1287093	zrkmbgw
40	1375890	xadovwz

*** CODING TASK***

Scripter: the coding task should be included here (see description above in Info3+4+5+6). First there are 2 codes to practice. Then the real task starts, and respondents have 8 minutes to solve the codes, alternatively they can also click the button 'Take a break' in this case they cannot solve any codes but you should time how long they take a break as this also adds to the points they earn. When taking a break, the time continues running.

Show Coding table (see image coding_table1.jpg untill coding_table40.jpg) + a code to translate in to numbers. Show an answer box where they can type the answer.

SCRIPTER: foresee 40 codes – respondents are to continue until the 6 minutes timer runs out.

If answer is correct go to next code, if answer is wrong show error message and they can try again: "Your answer is incorrect. Please try again."
For each code, a respondent can give 3x a wrong answer, then move to the next code. For this 3rd attempt, show error message: "Your answer is incorrect. The correct answer is: <insert correct answer of that code>. You can now proceed to the next code".

There should be a button "Switch to automatic mode", when this button is clicked it is not possible to do the coding task. During the break the button should change to "Switch to work mode" so they can start again (they can take several breaks if they want, during this 6 minute period). For Amount2 >0: if participants click on the "Switch to automatic mode" button, the amount donated shall increase by amount3 for every 10 seconds of break.

If Amount2 =0 On each coding screen, also show a graph with number of tasks they have already solved correctly.
If Amount2 >0 the graph should comprise a count of the monetary donation that had been collected up to this point by solving codes and/or taking a break.

Calculate hidden variables:
Scripter: count number of seconds respondent takes a break → variable: **CountSecBreak**.

Ipsos

Scripter: calculate **CountSecBreak2= CountSecBreak/10** and rounded down to whole number (e.g if CountSecBreak=306 → CountSecBreak2=30) – this *Amount3 is the number of points donated because of taking a break.

Scripter: count number of tasks coded correctly → variable: **CountCodingCorrect**.

Scripter: calculate **AmountDonated** = number CountCodingCorrect *<Amount2> + CountSecBreak2*<Amount3>

Scripter: if treatment=5, 9, 10 or 11 calculate **AmountEarned**= number CountCodingCorrect *10 points

Calculate

- AmountDonated2=for country=1 (IT): AmountDonated/100 (=amount in EUR) OR for country =2 (PL) AmountDonated*0.026 (=amount in złoty)
- AmountEarned2=for country=1 (IT): AmountEarned/100 (=amount in EUR) OR for country =2 (PL) AmountEarned*0.026 (=amount in złoty)
- AmountSanction2 =for country=1 (IT): AmountSanction/100 (=amount in EUR) OR for country =2 (PL) AmountSanction*0.026 (=amount in złoty)

Base: all respondents

Info21 [infoscreen]

You have solved **<CountCodingCorrect> tasks** and took **<CountSecBreak> seconds of break** in 6 minutes.

If Treatment=2, 3, 4, 7, 8 add: [Amount2 != 0]

If **AmountDonated** >0:

This results in a **donation to <if country=1: Banco Alimentare, if country=2: Banki Żywqności> of <AmountDonated>** (CountCodingCorrect *<Amount2> points for solving tasks **+** CountSecBreak2*<Amount3> points generated in the automation mode). Thank you for your contribution!

If **AmountDonated** =0:

There will be no donations to **<if country=1: Banco Alimentare, if country=2: Banki Żywności>.**

If Treatment=5, 9, 10, 11, add: [Treatment = MonReward]

If **AmountDonated** >0:

This results in a **donation to <if country=1: Banco Alimentare, if country=2: Banki Żywności> of <AmountDonated>** (CountCodingCorrect *<Amount2> points for solving tasks **+** CountSecBreak2*<Amount3> points generated in the automation mode). Thank you for your contribution!

Additionally, **you receive <AmountEarned>** in excess of your participation fee for solving the <CountCodingCorrect> tasks.

If **AmountDonated** =0:

There will be no donations to **<if country=1: Banco Alimentare, if country=2: Banki Żywności>.**

You do not receive any points in excess of your participation fee since you solved 0 tasks correctly.

Base: all respondents

Q9 [S]

To conclude part I of this study and to briefly follow up on the recent task, please indicate, **how difficult was it for you to solve the numerical codes in Task I**?

1. Very difficult
2. Fairly difficult
3. Neither difficult nor easy
4. Fairly easy
5. Very easy
98. Don't know

Base: all respondents

Q9b [S]

And how exhausting was it for you to solve the numerical codes in Task I?

1. Very exhausting
2. Fairly exhausting
3. Neither exhausting nor easy
4. Fairly easy
5. Very easy
98. Don't know

Filler task

Base: all respondents

Info22 [infoscreen]*Scripter: if treatment 1-5 or 7-11:*

From this page on, part II of the study starts.

In the following, we would like you to answer a set of questions. To reimburse you for your effort in answering these questions, **you receive additional survey points amounting to <Amount1>**. Please note, this **payoff does NOT depend on whether you answer the questions correctly**, nor in which time you answer the questions correctly.

Scripter: The purpose of the bonus payment is to assure a similar endowments of participants before conducting Task II. The challenge is to equalize the payoff of participants in the "MonReward" treatment and the other treatments. Thus, in the "MonReward" treatment, the additional payoff will be small at X€. In the other treatments, the distribution of these payoffs should resemble the additional payments, participants obtained if they had been receiving a reward in Task I. Therefore, the payment for the filler task in all but the "MonReward" treatment should be distributed equivalently to the distribution of additional payoff participants made in the treatment "MonReward" + the amount X€.

Scripter: Assign Amount5 according to table, i.e. depending on gender, education, age, and device

ID: [Female=1, Male=0], [(Education>=16 years)=1, (Education<16 years)=0], [(Age<=40)=1, (Age>40)=0], [Laptop/PC=1, Laptop/PC=0]

Education>=16 if “wyższe licencjackie lub zawodowe (bez magisterium)”, "Diploma universitario / extra-universitario", "Laurea di primo livello/laurea triennale","Diploma di laurea (vecchio ordinamento)", "Laurea specialistica a ciclo unico", "Laurea di secondo livello/laurea specialistica", "wyższe magisterskie (Magister)", "studia doktoranckie (Doktor)"

Scripter: Create variable **ID_Amount5**
ID_Amount5=1 IF gender = 1 and ISCED < 3 and age > 40 and Sniffer_device_type_initial > 1
ID_Amount5=2 IF gender = 1 and ISCED < 3 and age > 40 and Sniffer_device_type_initial = 1
ID_Amount5=3 IF gender = 1 and ISCED < 3 and age <41 and Sniffer_device_type_initial > 1
ID_Amount5=4 IF gender = 1 and ISCED < 3 and age <41 and Sniffer_device_type_initial = 1
ID_Amount5=5 IF gender = 1 and ISCED = 3 and age > 40 and Sniffer_device_type_initial > 1
ID_Amount5=6 IF gender = 1 and ISCED = 3 and age > 40 and Sniffer_device_type_initial = 1
ID_Amount5=7 IF gender = 1 and ISCED = 3 and age <41 and Sniffer_device_type_initial > 1
ID_Amount5=8 IF gender = 1 and ISCED = 3 and age <41 and Sniffer_device_type_initial = 1
ID_Amount5=9 IF gender = 2 and ISCED < 3 and age > 40 and Sniffer_device_type_initial > 1
ID_Amount5=10 IF gender = 2 and ISCED < 3 and age > 40 and Sniffer_device_type_initial = 1
ID_Amount5=11 IF gender = 2 and ISCED < 3 and age <41 and Sniffer_device_type_initial > 1
ID_Amount5=12 IF gender = 2 and ISCED < 3 and age <41 and Sniffer_device_type_initial = 1
ID_Amount5=13 IF gender = 2 and ISCED = 3 and age > 40 and Sniffer_device_type_initial > 1
ID_Amount5=14 IF gender = 2 and ISCED = 3 and age > 40 and Sniffer_device_type_initial = 1
ID_Amount5=15 IF gender = 2 and ISCED = 3 and age <41 and Sniffer_device_type_initial > 1
ID_Amount5=16 IF gender = 2 and ISCED = 3 and age <41 and Sniffer_device_type_initial = 1

Labels ID_Amount5:
1 "male / education <16 years / age > 40 / Other device"
2 "male / education <16 years / age > 40 / Laptop/PC"
3 "male / education <16 years / age <=40 / Other device"
4 "male / education <16 years / age <=40 / Laptop/PC"
5 "male / education >=16years / age > 40 / Other device"
6 "male / education >=16years / age > 40 / Laptop/PC"
7 "male / education >=16years / age <=40 / Other device"
8 "male / education >=16years / age <=40 / Laptop/PC"
9 "female / education <16 years / age > 40 / Other device"
10 "female / education <16 years / age > 40 / Laptop/PC"
11 "female / education <16 years / age <=40 / Other device"
12 "female / education <16 years / age <=40 / Laptop/PC"
13 "female / education >=16years / age > 40 / Other device"
14 "female / education >=16years / age > 40 / Laptop/PC"
15 "female / education >=16years / age <=40 / Other device" 16 "female / education >=16years / age <=40 / Laptop/PC" Amount5:

ID	ID_Amount5	70	80	90	100	110	120	130	140	150	160	170	180	190	
[1] 0 0 0 0	1	0.4899	0.155	0.1084	0.1019	0.0723	0.0523	0.019	0.0009	0.0001	0.0001	0.0001			
[1] 0 0 0 1	2	0.2785	0.1673	0.1346	0.1103	0.0914	0.0683	0.0523	0.0432	0.0381	0.0154	0.0006			
[1] 0 0 1 0	3	0.2209	0.1085	0.1085	0.0963	0.1255	0.1228	0.0576	0.0552	0.0377	0.0132	0.017	0.013	0.0163	0
[1] 0 0 1 1	4	0.1133	0.0635	0.0967	0.0872	0.0549	0.0333	0.0708	0.1214	0.142	0.1115	0.0469	0.0405	0.0168	0
[1] 0 1 0 0	5	0.3941	0.0435	0.0835	0.1269	0.0691	0.0563	0.0455	0.0549	0.0451	0.0554	0.0238	0.0019		
[1] 0 1 0 1	6	0.245	0.0603	0.1305	0.1551	0.0634	0.0403	0.0526	0.0918	0.0509	0.0488	0.0475	0.0126	0.0012	
[1] 0 1 1 0	7	0.0298	0.0676	0.081	0.1337	0.0645	0.0891	0.2179	0.1539	0.0351	0.0014	0.0001	0.0019	0.0313	0
[1] 0 1 1 1	8	0.0218	0.0434	0.018	0.0175	0.0449	0.0239	0.0632	0.2062	0.254	0.1235	0.025	0.0026	0.0196	
[1] 1 0 0 0	9	0.4253	0.1064	0.1334	0.1199	0.1116	0.0602	0.0248	0.0141	0.0041	0.0001	0.0001			
[1] 1 0 0 1	10	0.1658	0.1081	0.1074	0.1264	0.0992	0.055	0.0502	0.0607	0.0505	0.0523	0.0388	0.04	0.0267	0
[1] 1 0 1 0	11	0.1616	0.0995	0.0558	0.0727	0.117	0.1319	0.1093	0.086	0.0619	0.0394	0.0331	0.0244	0.0072	0
[1] 1 0 1 1	12	0.0001	0.0007	0.0191	0.0671	0.1172	0.149	0.1539	0.129	0.1514	0.1322	0.0635	0.0164	0.0004	
[1] 1 1 0 0	13	0.4225	0.128	0.0828	0.0734	0.0966	0.0737	0.0487	0.0268	0.0338	0.0125	0.0012			
[1] 1 1 0 1	14	0.1255	0.1493	0.1757	0.0988	0.0691	0.0738	0.0976	0.0684	0.0283	0.0492	0.0477	0.0153	0.0013	
[1] 1 1 1 0	15	0.0689	0.0805	0.0648	0.1251	0.1537	0.1704	0.0957	0.0753	0.0758	0.0275	0.0139	0.0332	0.014	
[1] 1 1 1 1	16	0.0491	0.0266	0.0335	0.028	0.0389	0.0482	0.0882	0.1054	0.1254	0.0947	0.084	0.0435	0.0454	0

**Treatment**	**Amount1**
2	Amount5
3	Amount5
4	Amount5
5	<if **CountCodingCorrect<7:**<br>**(7- CountCodingCorrect)*10 points>**
9	<if **CountCodingCorrect<7:**<br>**+ (7- CountCodingCorrect)*10 points>**

10	<if **CountCodingCorrect<7:**<br>**+ (7- CountCodingCorrect)*10 points>**

Base: all respondents

Q10a [Q] (range 0-99)

A bat and a ball cost 1.10<if country=1: €, if country=2: złoty> in total. The bat costs 1.00<if country=1: €, if country=2: złoty> more than the ball.
How much does the ball cost?

______ cents

(correct answer: 5)

Base: all respondents

Q10b [Q] (range 0-100)

If it takes 5 machines and 5 minutes to make 5 widgets, how long would it take 100 machines to make 100 widgets?

______ minutes

(correct answer: 5)

Base: all respondents

Q10c [Q] (range 0-99)

An expedition on a mountain climbing trip was traveling with eleven horse packs. Each horse can carry only three packs. How many horses does the expedition need?

______ horses

(correct answer: 4)

Base: all respondents

Q10 [Q] (range 0-99)

In a lake, there is a patch of lily pads. Every day, the patch doubles in size. If it takes 48 days for the patch to cover the entire lake, how long would it take for the patch to cover half of the lake?

______ day

(correct answer: 47)

Base: all respondents

Q11 [Q] (range 0-99)

If you are running a race and you pass the person in second place, what place are you in?

Place: _____

(correct answer: second)

Base: all respondents

Q12 [Q] (range 0-99)

A farmer had 15 sheep and all but 8 died. How many are left?

_____

(correct answer: 8)

Base: all respondents

Q13 [Q] (range 0-999)

How many cubic meters of dirt are there in a hole that is 3m deep x 3m wide x 3m long?

________ cubic meters

(correct answer: none)

Base: all respondents

Q14 [SGRID - progressive grid]

To what extent do you agree, or disagree, with the following statements?

Rows (randomize)

1. I have little control over the things that happen to me
2. There is really no way I can solve some of the problems I have
3. There is little I can do to change many of the important things in my life
4. I often feel helpless in dealing with the problems of life
5. Sometimes I feel that I'm being pushed around in life
6. What happens to me in the future mostly depends on me
7. I can do just about anything if I really set my mind to it

Columns

1. Strongly agree
2. Tend to agree
3. Neither agree nor disagree
4. Tend to disagree
5. Strongly disagree

98. Don't know

TASK II – Donation decision

Base: all respondents

Q15 [drop down list + Q] (range 0-99999)
You have gained **<AmountEarned + Amount1>** additional survey points.

We offer you the **opportunity to use a part of your additional survey points to donate** to the organizations listed below. **You can donate from 0 up to 70 points.** The amount you donate will be deducted from your collected additional points.

If you decide to donate to one of the organizations, **we will double your donation** by donating the same amount to the same charity/charities out of our own pocket.

If you want to take this donation opportunity, please select the charity you want to support from the drop down list and add the amount you would like to donate. The following organizations are available for selection. If you do not want to take this donation opportunity you may skip this question.

1. Donation to <insert drop down list with charities>: ________points
2. Donation to <insert drop down list with charities>: ________points
3. Donation to <insert drop down list with charities>: ________points

Scripter: add check that Sum does not exceed 70 points

<drop down list of charities>:

1. SolarAid
2. <if country=1: Italian Red Cross, if country=2: Polish Red Cross>
3. Doctors Without Borders
4. Save the children
5. World Wide Fund for Nature

Scripters: Use the following charities and description. Randomize the order of the charities.

SolarAid	**SolarAid**<br>An international development charity which is working to create a sustainable market for solar lights in Africa. In line with the Sustainable Development Goal 7: "Ensure access to affordable, reliable, sustainable and modern energy for all", the organisation's aim is to reduce global poverty and climate change through providing access to solar lights for rural communities.

	**<if country=1: Italian Red Cross, if country=2: Polish Red Cross>**<br>The <if country=1: Italian Red Cross, if country=2: Polish Red Cross> is the <if country=1: Italian, if country=2: Polish> body of the worldwide neutral and impartial humanitarian network the International Red Cross and Red Crescent Movement. At the heart of their work is providing help to people in crisis and in need, both in the <if country=1: Italian, if country=2: Polish> and overseas.
	**Doctors Without Borders**<br>An international humanitarian medical non-governmental organisation best known for its projects in conflict zones and in countries affected by endemic diseases. Main areas of work include diabetes, drug-resistant infections, HIV/AIDS, hepatitis C, tropical and neglected diseases, tuberculosis, vaccines and COVID. They contribute to patents and intellectual property subjects, as well as research and development
	**Save the children**<br>Save the Children was established to improve the lives of children through better education, health care, and economic opportunities, as well as providing emergency aid in natural disasters, war, and other conflicts.
	**World Wide Fund for Nature**<br>The World Wide Fund for Nature is an international non-governmental organization that works in the field of wilderness preservation and the reduction of human impact on the environment. WWF aims to stop the degradation of the planet's natural environment and to build a future in which humans live in harmony with nature.

POST QUESTIONNAIRE

Base: all respondents

Info23 [infoscreen]

As a last step, we kindly ask you to fill in a set of questions before the end of this study.

Base: all respondents

Info24

Please remember the task in part I. An **average person** in the task can decide themselves how **<if Amount2=0>** much effort to provide to solve the codes (see Table below).**<if Amount2>0>** much s/he wants to contribute to <if country=1: Banco Alimentare, if country=2: Banki Żywności> by **providing more or less effort** (see Table below).

Scripter: use amounts from task1 for values of XY_low, XY_medium, XY_high.

	Codes solved	Donation Scripter: Skip column if Amount2=0
Maximum effort	13	<XY_high>
Medium effort	10	<XY_medium>
Minimal effort	0	<XY_low>

Scripter: Randomize order of the next two questions (Q17+ Q18) + add variable that captures the order in which they are shown.

Scripter: always show the first sentence in question 2 independent of randomization of question 1 and 2 (so don't show for the question shown first)

Base: IF Treatment <> 4 AND IF Treatment <> 10 (not for Social Norm Nudge) and Treatment<>1

Q16 [S - slider]

Scripter: If Q18 is shown fist, add this first sentence: Following on from the previous question. We kindly ask you to guess which of the following numbers of codes **other participants rated as the appropriate** number to solve in the task, independent of your own opinion on the appropriate behavior. "Appropriate" behavior means the behavior that **others consider to be "correct" or "moral"**. The standard is, hence, the opinion of others, independently of your personal opinion.

We kindly ask you to answer as precisely as possible. If your **guess is correct, you will gain 10 points. If your guess is incorrect, you will not gain any additional points for your guess.**

I think that other participants rated on average the appropriate number of solved codes for this task to be: Scripter: insert slider with left anchor: XY_Low and right anchor XY_high.

Give points to respondents depending on correct value (see table below) and their answer.
If respondent's answer=Correct value, award 10 points.

**Correct value**
11 codes

Scripter: insert slider with left anchor: 0 and right anchor 25

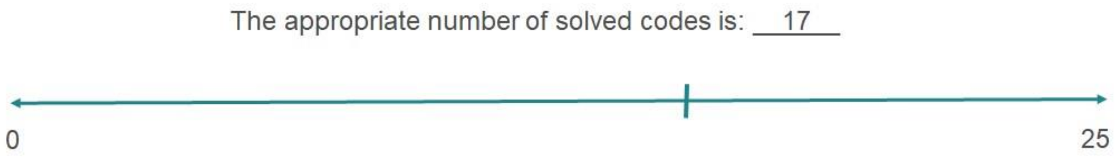

Low effort Medium effort High effort

Base: IF Treatment <> 3 AND IF Treatment <> 9 (not for Personal Norm Nudge) and Treatment<>1

Q17 [S - slider]

Scripter: If Q17 is shown fist, add this first sentence: Following on from the previous question. We would like you to evaluate, **according to your own opinion** and independently of the opinion of others, which of the following numbers of codes would be **appropriate** to solve in the task. “Appropriate” behavior means the behavior that you **personally consider to be “correct” or “moral”**. The standard is, hence, your personal opinion, independently of the opinion of others.

We kindly ask you to answer as precisely as possible with your own honest opinion. There is **no right or wrong answer**; you will not get any additional points depending on your answer.

I think that the appropriate number of solved codes for this task would be:

Scripter insert slider with left anchor: 0 codes and right anchor 25, also show the selected amount

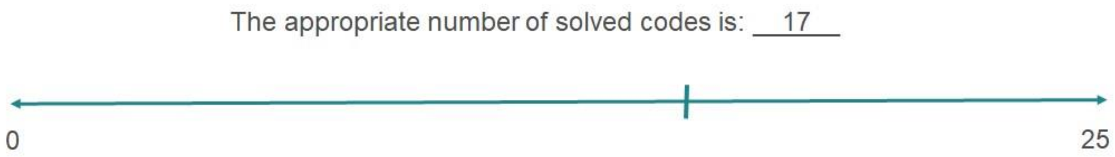

Low effort Medium effort High effort

Base: **if Amount2>0**

Q18 [S]

How much do you agree, or disagree, with the following statement:

Donating to **<if country=1: Banco Alimentare, if country=2: Banki Żywności>** is a reasonable measure to improve the wellbeing of others.

1. Strongly agree
2. Tend to agree
3. Neither agree nor disagree
4. Tend to disagree
5. Strongly disagree

98. Don’t know

Base: all respondents

Q18b [S]

How much do you agree, or disagree, with the following statement:

I had the notion that making moral/prosocial decisions in this study was expected from me.

1. Strongly agree
2. Tend to agree
3. Neither agree nor disagree
4. Tend to disagree
5. Strongly disagree
98. Don't know

Base: all respondents

Q20 [SGRID - progressive grid]

How much do you agree, or disagree, with the following statements?

Rows (randomize):

1. In general, people can be trusted.
2. Nowadays you can no longer rely on anyone.
3. When dealing with strangers, it is better to be cautious before trusting them.

Columns:

1. Strongly agree
2. Tend to agree
3. Neither agree nor disagree
4. Tend to disagree
5. Strongly disagree
98. Don't know

Base: all respondents

Q21 [SGRID - progressive grid]

How much trust do you have in the following institutions?

Rows (randomize):

1. The European Union
2. if Country=1: Italy's Government if country=2: Poland's Government
3. Scientists (e.g. research institutes, universities, academics)
4. Media (e.g., press, public broadcasters)

Columns:

1. Strongly distrust
2. Tend to distrust
3. Neither trust nor distrust
4. Tend to trust

5. Strongly trust
98. Do not know / not applicable

Base: all respondents

Q22 [S]

How much do you agree, or disagree, with the following statement: In general, there are many people who engage in prosocial activities, like volunteering, donations to charity, engagement in an NGO etc.

1. Strongly agree
2. Tend to agree
3. Neither agree nor disagree
4. Tend to disagree
5. Strongly disagree

Base: all respondents

Q23 [S]

Please indicate , in general, how willing or unwilling you are to take risks. Please use a scale from 0 to 10, where 0 means "completely unwilling to take risks" and a 10 means you are "very willing to take risks". You can also use any numbers between 0 and 10 to indicate where you fall on the scale, like 0, 1, 2, 3, 4, 5, 6, 7, 8, 9, 10.

0. 0 - Fully risk avers (not willing to take any risks)
1. 1
2. 2
3. 3
4. 4
5. 5
6. 6
7. 7
8. 8
9. 9
10. 10 - Fully risk seeking (very willing to take risks)

Base: all respondents

Q24 [S]

Have you made any donations to charity/ies in the past year?

1. Yes
2. No

Base: IF Q24=1

Q25 [S] How much have you donated in total approximately over the course of the last year?

1.Less than if country=1: 10€ if country=2:26 Zł

2.If country=1: 10-50€, If country=2: 26-130 Zł

3.More than If country=1: 50€, If country=2:130 Zł

Base: all respondents

Q26 [S]

How willing are you to give up something that is beneficial for you today in order to benefit more from that in the future?

1. Very unwilling
2. Unwilling
3. Neither willing nor unwilling
4. Willing
5. Very willing
98. Don’t know
99. Prefer not to say

Base: all respondents

Q27 [SGRID - progressive grid]

To what extent do the following statements apply to you?

Rows (randomize)

1. I usually turn the light(s) off when I am leaving a room
2. I usually install energy efficient appliances at home
3. I only use the washing machine when I have a full load
4. I recently insulated or aim to insulate my house in the near future

Columns

1. Fully applies
2. Rather applies
3. Neither it applies nor it doesn’t
4. Rather not applies
5. Not applies at all
98. Don’t know

Base: all respondents

Q28 [SGRID - progressive grid]

Please indicate whether the following statements apply to you or not.

Rows (randomize)

1. I have contacted or aimed to contact my energy retailer to negotiate a better deal on my energy bill in the last three years
2. I have contacted or aimed to contact a consumer support association to complain and/or to resolve a dispute in the last three years
3. I usually ask or aim to ask advice on my energy bill to my friends

Columns

1. Fully applies
2. Rather applies
3. Neither it applies nor it doesn’t
4. Rather not applies
5. Not applies at all
98. Don’t know

Base: all respondents

Q29 [SGRID - progressive grid]

Please indicate whether the following statements apply to you or not.

Rows (randomize)

1. I am a member of an energy community
2. I produce my own energy
3. I am willing to support vulnerable citizens, who cannot pay their energy bills

Columns

1. Fully applies
2. Rather applies
3. Neither it applies nor it doesn’t
4. Rather not applies
5. Not applies at all
98. Don’t know

Base: all respondents

Q30 [S]

Did you recently apply for / benefit from income support schemes (e.g. to pay energy bills, etc.)?

1. Yes
2. No
98. Don’t know
99. Prefer not to say

SOCIO-DEMOGRAPHICS

Base: all respondents

SD1 [S]

What is your mother tongue?

1. if country=1, show: Italian if country=2, show: Polish

98. Other

Base: all respondents

SD2 [S]

The next question is related to your political opinion. A "Prefer not to answer" option is available for you to select, at your discretion. Collecting such information enables us to understand how attitudes are related to certain behaviours. Participation is always voluntary, and your responses are used for research purposes only, combined with the answers from all other participants. We will provide our client only anonymous, aggregated results. The data will be held for no longer than 12 months. Do you accept the collection of this data?

1. Yes
2. No

Base: if SD2=1

SD3 [S]

Which party would you vote for if there were a federal election next Sunday?

Scripter: if country = 1 (Italy) randomize items 1-5

1. Fratelli d'Italia
2. Forza Italia
3. Partito Democratico
4. Movimento Cinque Stelle
5. Lega
6. Others (please specify) [O]

Scripter: if country = 2 (Poland) randomize items 1-5

7. Prawo i Sprawiedliwość
8. Polskie Stronnictwo Ludowe
9. Koalicja Obywatelska
10. Sojusz Lewicy Demokratycznej
11. Konfederacka Wolność i Niepodległość
12. Others (please specify) [O]

ALL COUNTRIES

98. Don't know

99. Prefer not to answer

Base: all respondents

SD4 [S]

Could you please indicate your household's monthly income (that is, after income taxes have been paid)?

Your total household income includes your own income plus the incomes of all household members who live together with you. The total income includes income from jobs, pensions, social security, interest, dividends, capital gains claimed, profits from businesses, unemployment payments, and all other money you received.

Scripter: if country = 1 (Italy)
1. less than 600 euro
2. between 600 and 849 euro
3. between 850 and 1049 euro
4. between 1050 and 1249 euro
5. between 1250 and 1449 euro
6. between 1450 and 1649 euro
7. between 1650 and 1899 euro
8. between 1900 and 2199 euro
9. between 2200 and 2799 euro
10. 2800 euro or more

Scripter: if country = 2 (Poland)
11. less than 1300 złoty
12. between 1300 and 1649 złoty
13. between 1650 and 1949 złoty
14. between 1950 and 2249 złoty
15. between 2250 and 2549 złoty
16. between 2550 and 2849 złoty
17. between 2850 and 3199 złoty
18. between 3200 and 3699 złoty
19. between 3700 and 4599 złoty
20. 4600 złoty or more

ALL COUNTRIES
98. Don't know
99. Prefer not to answer

Base: all respondents

Thank you very much for taking part in this survey.

If Amount2=0, add:

Thanks to your participation we will support charity by donating <AmountCharity> points to the charity of your choice (this corresponds to <AmountCharity2> <if country=1: €, if country=2: Zł>).

If Amount2>0, add:

Thanks to your participation we will support charity by donating < AmountDonated > points to <if country=1: Banco Alimentare, if country=2: Banki Żywności> (this corresponds to < AmountDonated2> <if country=1: €, if country=2: Zł>), and <AmountCharity> points to the charity of your choice (<AmountCharity2> <if country=1: €, if country=2: Zł>).

if PointsToPay > 0, and Supplier=1100 (iSay), add:
You have earned <PointsToPay> additional points in this survey (this corresponds to <PointsToPay2> <if country=1: €, if country=2: Zł>). The equivalent of this amount will be paid

via your panel-account in your usual points. <PointsToPay3> points will be added to your account after the survey is closed (note that this may take a while).

if PointsToPay > 0, and SupplierID=10302, add:
You have earned <PointsToPay> additional points in this survey (this corresponds to <PointsToPay2> <if country=1: €, if country=2: Zł>). This amount will added to your account after the survey is closed (note that this may take a while).

Show all:
Wishing you a nice day.

Scripter: calculate : PointsToPay= AmountEarned + Amount1 + Q2_earned + Q4_earned + Q16_earned – Q15_1 – Q15_2 – Q15_3

PointsToPay2=for country=1 (IT): PointsToPay/100 (=amount in EUR) OR for country =2 (PL) PointsToPay*0,026 (=amount in złoty)

PointsToPay3=for country=1 (IT): PointsToPay/0.9 (=amount in Ipsos Points) OR for country =2 (PL) PointsToPay/0.6*0,0055556 (to take into account the EXCHANGE RATE) (=amount in Ipsos Points)

AmountCharity = (Q15_1 + Q15_2 + Q15_3)*2 (the amount donated in Q15 – doubled)

AmountCharity2 = for country=1 (IT): AmountCharity /100 (=amount in EUR) OR for country =2 (PL) AmountCharity*0,026 (=amount in złoty)

References

Picard, J. (2023). Double-edged nudges? micro-foundations to behavioural interventions and their spillover effects. *Micro-Foundations To Behavioural Interventions and Their Spillover Effects (January 6, 2023).*

Tibshirani, R. (1996). Regression shrinkage and selection via the lasso. *Journal of the Royal Statistical Society Series B: Statistical Methodology*, 58(1):267–288.
